# Discontinuing cotrimoxazole preventive therapy in HIV-infected adults who are stable on antiretroviral treatment in Uganda (COSTOP): A randomised placebo controlled trial

**DOI:** 10.1371/journal.pone.0206907

**Published:** 2018-12-31

**Authors:** Zacchaeus Anywaine, Jonathan Levin, Ronnie Kasirye, Joseph Kayiira Lutaakome, Andrew Abaasa, Andrew Nunn, Heiner Grosskurth, Paula Munderi, on behalf of the COSTOP research team

**Affiliations:** 1 Medical Research Council / Uganda Virus Research Institute and London School of Hygiene and Tropical Medicine Uganda Research Unit, Entebbe, Uganda; 2 School of Public Health, University of Witwatersrand, Johannesburg, South Africa; 3 MRC Clinical Trials Unit at University College London, London, United Kingdom; 4 Department of Infectious Disease Epidemiology, London School of Hygiene and Tropical Medicine, London, United Kingdom; University of KwaZulu-Natal, SOUTH AFRICA

## Abstract

**Background:**

Cotrimoxazole (CTX) preventive therapy (CPT) reduces opportunistic infections and malaria in HIV-infected patients. In Africa, policies on sustained CPT during antiretroviral therapy (ART) differ between countries. We assessed the safety of discontinuing CPT in stable patients on ART in Uganda.

**Methods:**

COSTOP was a double-blind placebo-controlled trial. Patients aged ≥18 years, on CPT, and stable on ART (CD4 counts ≥250 cells/μL); were randomised to daily oral placebo (PLC group) or cotrimoxazole 960 mg/tablet (CTX group). Co-primary outcomes were: (i) time to first cotrimoxazole-preventable infection, with non- inferiority of PLC defined as the upper one-sided 95% confidence limit of the adjusted hazard ratio(aHR) ≤1.25; and (ii) time to first grade 3/4 haematological adverse event.

**Findings:**

2180 subjects (1091 PLC; 1089 CTX) were enrolled. 932 PLC and 943 CTX completed the trial after 12 months minimum follow up. Ninety-eight participants (59 PLC; 39 CTX) experienced 120 cotrimoxazole- preventable events, mainly bacterial pneumonia (72 events, 4 deaths PLC); (48 events, 2 deaths CTX). The aHR for time to first event was 1.57 (upper one-sided 95% confidence limit 2.21) in per protocol population (similar results in ITT population). 551 participants (318 CTX; 233 PLC) experienced 1043 haematological adverse events (616 CTX; 427 PLC). Time to the first adverse event, mainly neutropenia, was shorter in the CTX group (aHR 0.70 95%CI 0.59–0.82; log-rank χ2 = 18.08; P<0.0001). 362 (276 PLC, 86 CTX) participants experienced at least one episode of confirmed clinical malaria (P<0.0001).

**Interpretation:**

In ART stable patients with CD4 counts ≥250 cells/μL, continued CPT significantly reduces risk of severe bacterial infections and protects against malaria, while discontinuing CPT reduces haematological adverse events.

## Introduction

Primary cotrimoxazole preventive treatment (CPT) has been recommended as part of the minimum package of care for people living with HIV/AIDS (PLHIV) in Africa since 2001 [1], a practice supported by clinical trials among antiretroviral naïve patients with advanced HIV disease; which demonstrated that CPT significantly reduces HIV related mortality, bacterial infections, malaria and related hospital admissions.[2,3]

Subsequent to wide spread availability of antiretroviral therapy (ART), survival and immune function of PLHIV in low and middle income countries have significantly improved, [4] making the added value of sustained CPT alongside ART unclear. This is an important consideration since the CPT intervention represents a substantial increase in overall costs of HIV care programs. [5] In addition to placing a higher pill burden on ART treated patients, cotrimoxazole is associated with haematological toxicity and other unwanted effects such as allergic reactions, photosensitivity, interstitial nephritis and cross-resistance with some of the antimalarial drugs in frequent use.[6–9]

In high-income countries, primary CPT is not continued among patients on ART after CD4 count reconstitution to 200 cells/μL or once HIV viraemia becomes undetectable.[8,10] In low income countries, policies on CPT during ART differ; with a majority of national guidelines recommending discontinuation of CPT following CD4 count reconstitution thresholds that range from 200–500 cells/μL and some countries still recommending that CPT during ART be continued indefinitely.[11]

Based on observational studies [12–14] and unblinded randomised trials on discontinuing CPT among patients on ART,[15,16] the World Health Organisation (WHO) in 2014 issued a recommendation that CPT may be discontinued in adults on ART following HIV virologic suppression, but that cotrimoxazole prophylaxis should be continued in settings where bacterial infections and malaria are highly prevalent.[17] However, the safety and risk benefits of stopping CPT in ART stable patients in low and middle countries have not been assessed in a double blind randomised controlled trial.

We report on the results of a double blind placebo controlled trial in Uganda to investigate the safety of discontinuing CPT and potential benefits in reduced cotrimoxazole-associated toxicity. Information on the design of this trial and malaria-specific findings of discontinuing CPT have been reported previously [18,19].

## Methods

### Study design

The safety of discontinuing CPT was assessed by determining whether the experimental (placebo) group was non-inferior to the control group (in which CPT was continued), with respect to the incidence of cotrimoxazole (CTX) preventable infections. The potential benefits of discontinuing CPT were studied by comparing rates of haematological adverse events in both groups, testing for superiority of the placebo group. The trial was conducted in two research clinics of the MRC/UVRI and LSHTM Uganda Research Unit, located in a district hospital in Entebbe town and a regional referral hospital in Masaka town. We obtained approvals from the Research Ethics Committee of the Uganda Virus Research Institute, the Uganda National Council for Science and Technology and the Uganda National Drug Regulatory Authority. Details of the study protocol have been reported earlier[18,19] The trial was registered (ISRCTN44723643).

### Participants

Patients were recruited from HIV care centres situated near-by where they continued to receive their ART. They were eligible if aged ≥18 years (before protocol amendment of December 2011, age limit was 18 to 59 years), had been on ART and daily CPT for at least 6 months, had a confirmed CD4 count of ≥250 cells/μL, no contraindication to taking CTX and having provided informed consent. Patients were excluded if they had an acute illness, history of grade 3 or 4 anaemia, neutropenia or thrombocytopenia, were receiving secondary CPT for a previous opportunistic infection or in first trimester pregnancy. All participants gave written informed consent for screening procedures and for recruitment, with independently witnessed consent if not literate.

### Randomisation and masking

Patients were randomised 1:1 to receive placebo (PLC) or active CTX. They were requested to take trial drugs as prescribed, submit any previous CTX supply still in their possession, and not use CTX from other sources. A randomisation schedule was developed using random permuted blocks with varying block size. We used five different block sizes of 8, 10, 12, 14 and 16. Separate randomisations were performed in four strata defined by study site (Entebbe or Masaka) and baseline CD4 count (250–499 cells/μL or ≥500 cells/μL). Trial drugs (CTX or placebo) sufficient for the period between scheduled study visits, were packed by independent supervised assistants, using identical containers labelled with trial numbers that corresponded to the randomisation register. At enrolment, clinicians entered the details of each participant in the next available row of the enrolment register, stratified by CD4 count. The trial number corresponding to that row was used on all trial documents of that patient and to identify the pre-packed study medication. Participants and investigators remained blinded to treatment allocation throughout. Only an independent statistician had access to the randomisation codes. At the end of follow up, exit interviews were conducted to determine whether participants had adhered to the protocol with respect to their treatment allocation.[20]

### Procedures

Consenting patients were screened 2 to 4 weeks before enrolment. Their medical history was recorded, and a physical examination, full blood count, CD4 count and urine pregnancy test (in women of reproductive age) performed. At enrolment, all were requested to continue the ART regimen provided by their HIV care provider. They received pre-labelled study drug as described above, comprising one oral tablet of 960mg CTX daily (CTX group) or one oral tablet daily of a matching placebo (PLC group). Study drugs had been obtained from CIPLA Ltd India. For ethical reasons and to limit the confounding effect of an imbalance in the routine use of effective malaria preventive interventions, at enrolment and once during follow-up each patient was issued with an insecticide treated bed net (ITN) and instructed to sleep under it. Follow up assessments, conducted by study nurses and doctors were scheduled monthly for the first 3 months to assess health and adherence to medications and then 3-monthly thereafter. At each visit clinical information was recorded, and a blood slide examination for malaria parasites and full blood count performed. CD4 counts were performed every 6 months after enrolment. Patients received a new supply of study drug every 3 months. Adherence to study drug and to ART was assessed at each clinic visit using pill counts and self-report questionnaires, and reported ITN use recorded. Patients were also encouraged to report to their study clinic whenever they were ill. Enrolment took place between 20 January 2011 and 21 March 2013. Trial duration was three years (2011–2014). Patients were followed for a minimum of 12 and a maximum of 36 months. At study exit, all patients were prescribed open-label cotrimoxazole as prophylactic treatment in keeping with national guidelines and continued their usual ART.

### Outcomes

The two co-primary outcome measures were: (i) time to the first CTX-preventable event or CTX-preventable death and; (ii) time to the first grade 3 or 4 haematological adverse event. We defined cotrimoxazole preventable clinical events based on biological plausibility, as those infections listed in the WHO surveillance clinical classification of HIV-related disease in adults,[21] against which cotrimoxazole has known biological activity, (see S1 Appendix. Cotrimoxazole preventable events). An independent end-point review committee (ERC), who were blinded to treatment allocation, reviewed all reported clinical events and deaths and adjudicated whether these fulfilled WHO surveillance clinical staging definitive or presumptive diagnostic criteria[21] and whether they could be defined as a CTX-preventable event. Laboratory tests for haematologic toxicity were performed at the MRC/UVRI laboratories in Entebbe. Results were graded according to the USA National Institute of Allergy and Infectious Diseases—Division of AIDS severity grading table, [22] and referred for assessment to the ERC if associated with a significant clinical event such as septicaemia. Secondary outcomes included the incidence of all CTX-preventable events, all-cause mortality, hospitalisations, asymptomatic and symptomatic malaria episodes confirmed by a positive parasitaemia blood slide, serious adverse events, the mean changes in CD4 count and other haematological parameters 48 weeks after enrolment, as well as adherence to ART, trial drug and ITN use.

### Statistical analysis

The sample size was based on assumptions described elsewhere.[18] In brief the assumed rate of CTX-preventable clinical events in the control group would be 10 per 100 PYO, based on an analysis of event rates from the DART trial among participants with confirmed CD4 count above 250 cells/μL[23] and loss to follow up rate would be 4% per year[23]. Recruiting 2000 participants was expected to provide 80% power, at the 5% level of significance (one-sided for non-inferiority), to determine non-inferiority (upper limit of the one-sided 95% CI for the aHR <1.25) of CPT discontinuation with respect to cotrimoxazole-preventable clinical events. For the haematological co-primary end-point, the sample would have 80% power to detect a 50% reduction in the expected rate of grade 3 or 4 haematological events.

In May 2012 the trial steering committee and regulatory bodies permitted recruitment of an additional 180 participants in order to accommodate a sub-study on microbial translocation and immune activation in the COSTOP trial population (results to be reported elsewhere). Data were double-entered and verified in MS Access (Microsoft, Redmond, Washington, USA) and analysed using Stata 13 (Stata Corp, College Station, Texas, USA). Two data sets were generated for the efficacy analysis, i.e. for per protocol (PP) and intention to treat (ITT) populations. Demonstration of non-inferiority of the PLC group with respect to CTX-preventable events requires that it can be shown in both the primary PP analysis and in the ITT populations since non-adherers in the ITT population may dilute any real treatment related differences. On the other hand, the PP population may be prone to selection bias, particularly if loss to follow up was differential between trial arms with regard to the risk for CTX-preventable events.[24] The ITT data set comprised all subjects who took at least one dose of study drug and for whom follow-up was assessed at least once. Participants remained in the PP population as long as they reported taking at least 80% of trial drugs since their last visit. For the co-primary endpoint on haematological events and for all secondary endpoints, the analysis was based on the ITT population.

The non-inferiority of placebo to CTX with respect to CTX-preventable events would be demonstrated if the upper limit of the one-sided 95% confidence interval of the hazard ratio (HR) for placebo relative to CTX was no greater than 1.25 (a relative increase of 25%). In the event that non-inferiority was not shown, an analysis would be carried out on the ITT population to assess whether continuing CTX was superior to PLC with respect to CTX-preventable events. Superiority of placebo to CTX with respect to haematological adverse events would be demonstrated if there was a statistically significant reduction in time to occurrence of the first grade 3 or 4 haematological event. All time to event analyses were carried out by fitting Cox proportional hazards regression models adjusting for study site and CD4 stratum only. Changes in CD4 and other haematological parameters were carried out by fitting linear regression models to the week 48 result (subject to a transformation such as logarithmic or square root in the event of severe heteroscedasticity). Adjustment was made for the baseline result (similarly transformed if appropriate), study site and the actual number of days between the readings closest to baseline and week 48 respectively. Thus week 48 mean CD4, white blood cell (WBC) and neutrophil counts were subjected to a natural logarithmic transformation and week 48 mean platelet count to square root transformation. For each haematological parameter, its marginal mean was estimated from the fitted regression model with the baseline of the parameter and actual number of days between baseline and 48 week assessment set at their mean values, averaged over the two sites, and the parameter back-transformed (for example WBC was subject to an exponential transformation). An independent data monitoring committee (IDMC) conducted 5 interim analyses using the Peto-Haybittle approach during the trial, that is only a probability of equal to, or less than 0.001 at interim analysis would be considered grounds for recommending early stopping. On each occasion the IDMC recommended that the trial should continue as planned. As is standard when using the Peto-Haybittle approach [25], no adjustment was made to the significance level used for the final analysis.

## Results

**Fig 1:**
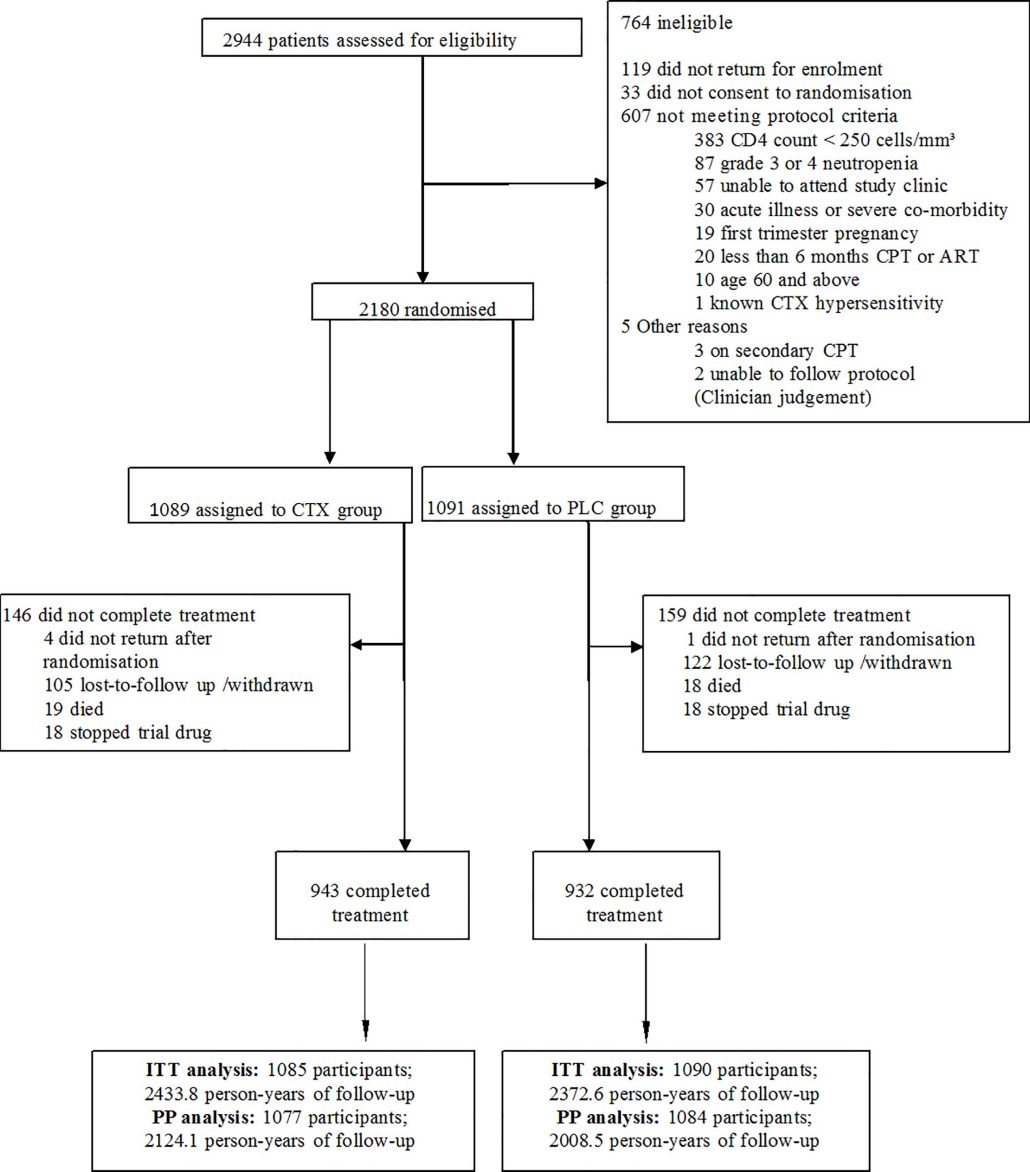
Trial profile.

A total of 2944 patients were screened, of whom 2180 (75%) were enrolled. Details of screen failures are presented in the trial profile (Fig 1). The main reasons for non-enrolment were low CD4 count, grade 3 or 4 neutropenia and failure to return after screening. Of the 2180 participants enrolled, 1089 were allocated to CTX and 1091 to PLC. Five patients did not return after enrolment. The remaining 2175 participants contributed 4806.4 person years of follow-up to the ITT analysis, and 1875 participants completed the trial. A total of 2161 participants contributed 4132.6 person years of follow-up to the PP analysis (Fig 1). For the ITT population, the median duration of follow-up was 2.3 years (IQR 1.2–2.8) for CTX and 2.1 years (IQR 1.1–2.8) for PLC participants. Baseline characteristics were similar between trial arms (Table 1). Slightly more participants were enrolled at Masaka (1178) than at Entebbe (1002). Almost 75% of participants were women, reflecting the gender distribution of patients registered at HIV care centres. About two thirds had been in WHO surveillance clinical stage 3 or 4 at the time of ART initiation. The median duration on ART was 47 months and median CD4 cell count at study entry was 518 cells/μL (IQR 410–688). Two thirds of participants reported that they slept under a mosquito net.

**Table 1:** Baseline characteristics.

Variable	Category	CTX (n = 1089)	Placebo (n = 1091)
Site	Entebbe	501 (46.0%)	501 (45.9%)
	Masaka	588 (54.0%)	590 (54.1%)
CD4 stratum	< 500	572 (52.5%)	570 (52.2%)
	≥ 500	517 (47.5%)	521 (47.8%)
CD4 at randomization	Median (IQR)	518 (410–696)	519 (411–682)
CD4 at randomization (grouped)	250–349	102 (9.4%)	142 (13.0%)
	350–499	414 (38.0%)	370 (33.9%)
	500 plus	573 (52.6%)	578 (53.0%)
Sex	Male	286 (26.3%)	283 (25.9%)
	Female	803 (73.7%)	808 (74.1%)
Age	Mean (s.d.)	41.0 (8.0)	40.7 (8.3)
	Median (IQR)	41 (36–46)	40 (35–47)
Weight (kg)	Mean (s.d.)	58.5 (10.7)	59.0 (10.8)
BMI (kg/m2)	Mean (s.d.)	22.2 (3.8)	22.5 (4.2)
WHO stage	I	54 (5.0%)	50 (4.6%)
	II	291 (26.9%)	303 (28.0%)
	III	621 (57.4%)	623 (57.6%)
	IV	115 (10.6%)	105 (9.7%)
Education level	None	111 (10.2%)	113 (10.4%)
	Primary	649 (59.6%)	660 (60.5%)
	Secondary	275 (25.3%)	268 (24.6%)
	University / vocational	53 (4.9%)	50 (4.6%)
Marital status	Married / cohabiting	473 (43.5%)	465 (42.6%)
	Separated etc.	576 (52.9%)	577 (52.9%)
	Single never married	39 (3.6%)	49 (4.5%)
Disclosed HIV status to partner	Yes	522 (85.6%)	520 (82.9%)
	No	88 (14.4%)	107 (17.1%)
Months on ART	Median (IQR)	48 (27–66)	47 (26–65)
Initial ART regimen	AZT / 3TC / NVP	521 (47.8%)	504 (46.2%)
	AZT / 3TC / EFZ	76 (7.0%)	74 (6.8%)
	d4T / 3TC / NVP	321 (29.5%)	348 (31.9%)
	d4T / 3TC / EFZ	33 (3.0%)	27 (2.5%)
	TDF / 3TC(FTC) / NVP	74 (6.8%)	73 (6.7%)
	TDF / 3TC(FTC) / EFZ	23 (2.1%)	25 (2.3%)
	AZT / 3TC / TDF	22 (2.0%)	31 (2.8%)
	Other	13 (1.2%)	7 (0.6%)
	Missing	6 (0.6%)	2 (0.2%)
Current regimen	AZT / 3TC / NVP	693 (63.6%)	716 (65.6%)
	AZT / 3TC / EFZ	131 (12.0%)	114 (10.4%)
	TDF / 3TC(FTC) / NVP	133 (12.2%)	134 (12.3%)
	TDF / 3TC(FTC) / EFZ	53 (4.9%)	50 (4.6%)
	AZT / 3TC / TDF	9 (0.8%)	8 (0.7%)
	Any PI	42 (3.9%)	44 (4.0%)
	Other	16 (1.5%)	16 (1.5%)
	Missing	12 (1.1%)	9 (0.8%)
Any ART switch prior to enrolment	Yes	497 (46.4%)	494 (45.7%)
When did you last miss ARVs?	Last week	17 (1.6%)	13 (1.2%)
	1–4 weeks ago	40 (3.7%)	50 (4.6%)
	1–3 months ago	41 (3.8%)	40 (3.7%)
	>3 months ago	133 (12.4%)	120 (11.1%)
	Never	842 (78.5%)	860 (79.4%)
Have you missed CTX in last month?	Yes	279 (25.7%)	232 (21.4%)
	No	808 (74.3%)	854 (78.6%)
Sleeps under a mosquito net	Yes	676 (62.1%)	694 (63.6%)

### Primary outcome (i)

**Fig 2:**
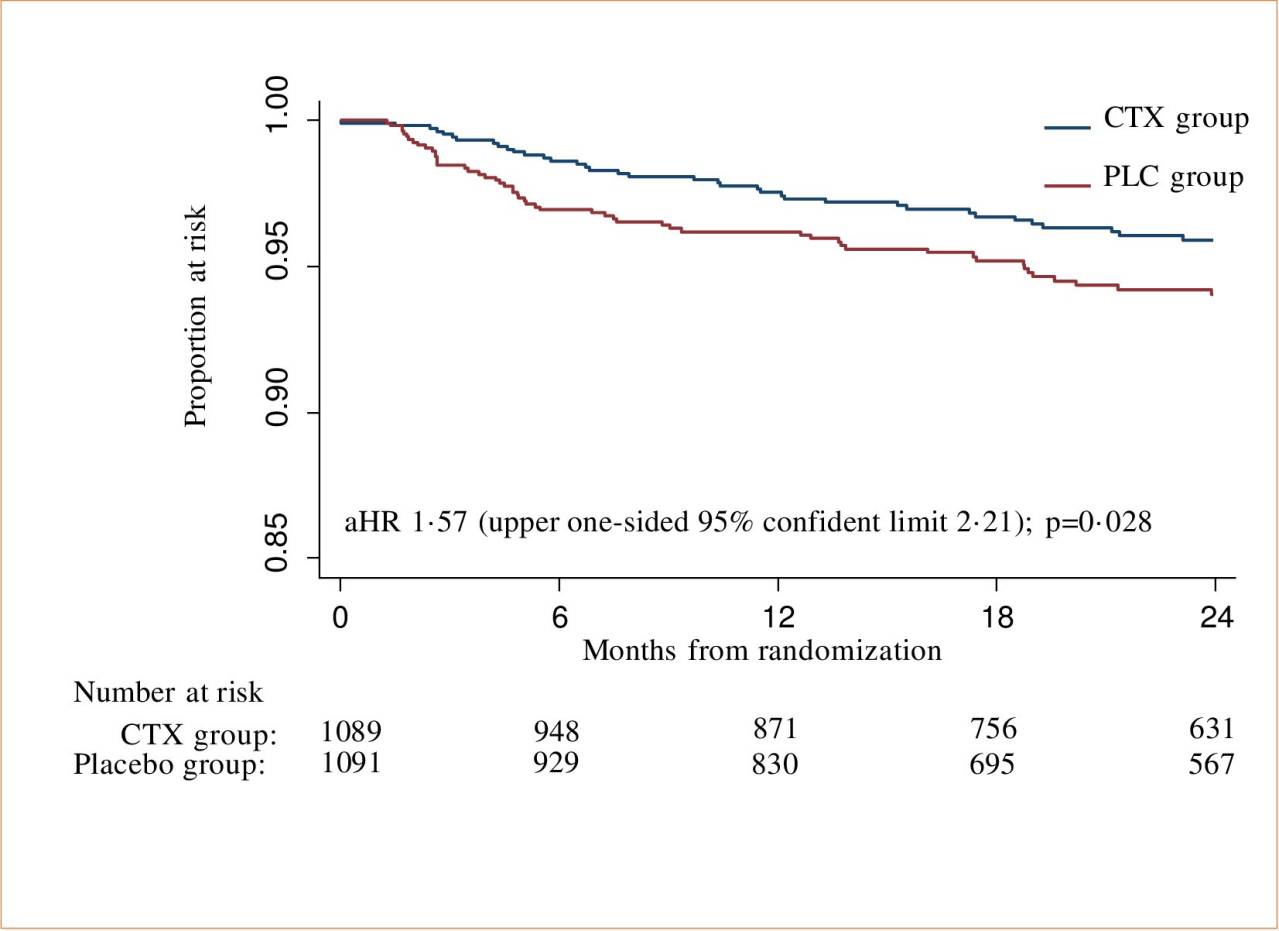
Time to first cotrimoxazole-preventable event–PP population.

In the PLC group 59 participants (5.4%) experienced a total of 72 events (including 4 deaths) compared to 39 (3.6%) participants in the CTX group who experienced a total of 48 events (including 2 deaths). Fig 2 shows a Kaplan-Meier plot of the time to the first CTX-preventable event by treatment group for the PP population (Fig 2). The results of a survival analysis for both the PP and ITT populations are summarized in Table 2, including the results of fitting a Cox regression model to the comparison of the two trial groups, adjusting for site and CD4 stratum. We failed to demonstrate non-inferiority of CPT discontinuation; in both the PP and ITT populations, the stratified log-rank test provided strong evidence that the risk of CTX-preventable events was higher in the PLC group than in the CTX group. For example, for the PP population the log-rank test was χ2 = 4.77; (P = 0.029). This was confirmed by the results of the Cox regression model: the adjusted hazard ratio (aHR) was 1.57 (upper one-sided 95% confidence limit 2.21) (Fig 2). The upper one-sided 95% confidence limit for the aHR of 2.21 exceeded the non-inferiority cut-off of 1.25. Similar results were obtained for the ITT population. There was some evidence (P = 0.057) of an interaction between CD4 count stratum and trial allocation. A sub-group analysis showed that the effect of CTX prophylaxis on CTX-preventable events was stronger in the higher CD4 count stratum (aHR 2.35; P = 0.006) than in the lower CD4 count stratum (aHR 1.07; P = 0.80) (Table 2). However, in both strata the upper one-sided 95% confidence limit for the aHR for PLC *vs*. CTX exceeded the non-inferiority cut-off of 1.25. The ITT analysis produced similar results. Based on these results, the computed number needed to treat over one year in order to prevent one WHO staging CTX-preventable clinical event is 113. The commonest non-fatal CTX-preventable events were bronchopneumonia (20 CTX, 33 PLC) and recurrent bacterial upper respiratory tract infections (5 CTX, 4 PLC). Categorisation of CTX-preventable events by treatment group is provided in a table (S1 Table. Non-fatal cotrimoxazole preventable events by treatment group).

**Table 2:** Survival analysis for the PP and ITT populations -number and rates of CTX-preventable events or deaths.

Analysis	Placebo group events	Placebo group Rate/100pyar (95% C.I.)	CTX group events	CTX group Rate/100pyar (95% C.I.)	Stratified log-rank statistic (P-value)	Adjusted Hazard Ratio (95% C.I.)*
Per protocol overall	59	2.94 (2.28; 3.79)	39	1.84 (1.34; 2.51)	4.77 (0.029)	1.57 (1.12; 2.21)
**Sub-group analysis by CD4 stratum**						
CD4<500cells/uL	25	2.4 (1.62; 3.55)	24	2.18 (1.46; 3.25)	0.07 (0.79)	1.07 (0.67; 1.72)
CD4≥ 500cells/uL	34	3.52 (2.51; 4.93)	15	1.46 (0.88; 2.43)	7.95 (0.005)	2.35 (1.41; 3.91)
ITT overall	59	2.49 (1.93; 3.21)	39	1.6 (1.17; 2.19)	4.5 (0.034)	1.55 (1.03; 2.32)
**Sub-group analysis by CD4 stratum**						
CD4<500cells/uL	25	2.03 (1.37; 3.00)	24	1.91 (1.28; 2.85)	0.04 (0.85)	1.06 (0.60; 1.86)
CD4≥ 500cells/uL	34	2.98 (2.13; 4.17)	15	1.27 (0.77; 2.11)	7.86 (0.005)	2.33 (1.27; 4.27)

* The upper limit of the CI gives the upper one-sided 95% confidence limit.

### Primary outcome (ii)

**Fig 3:**
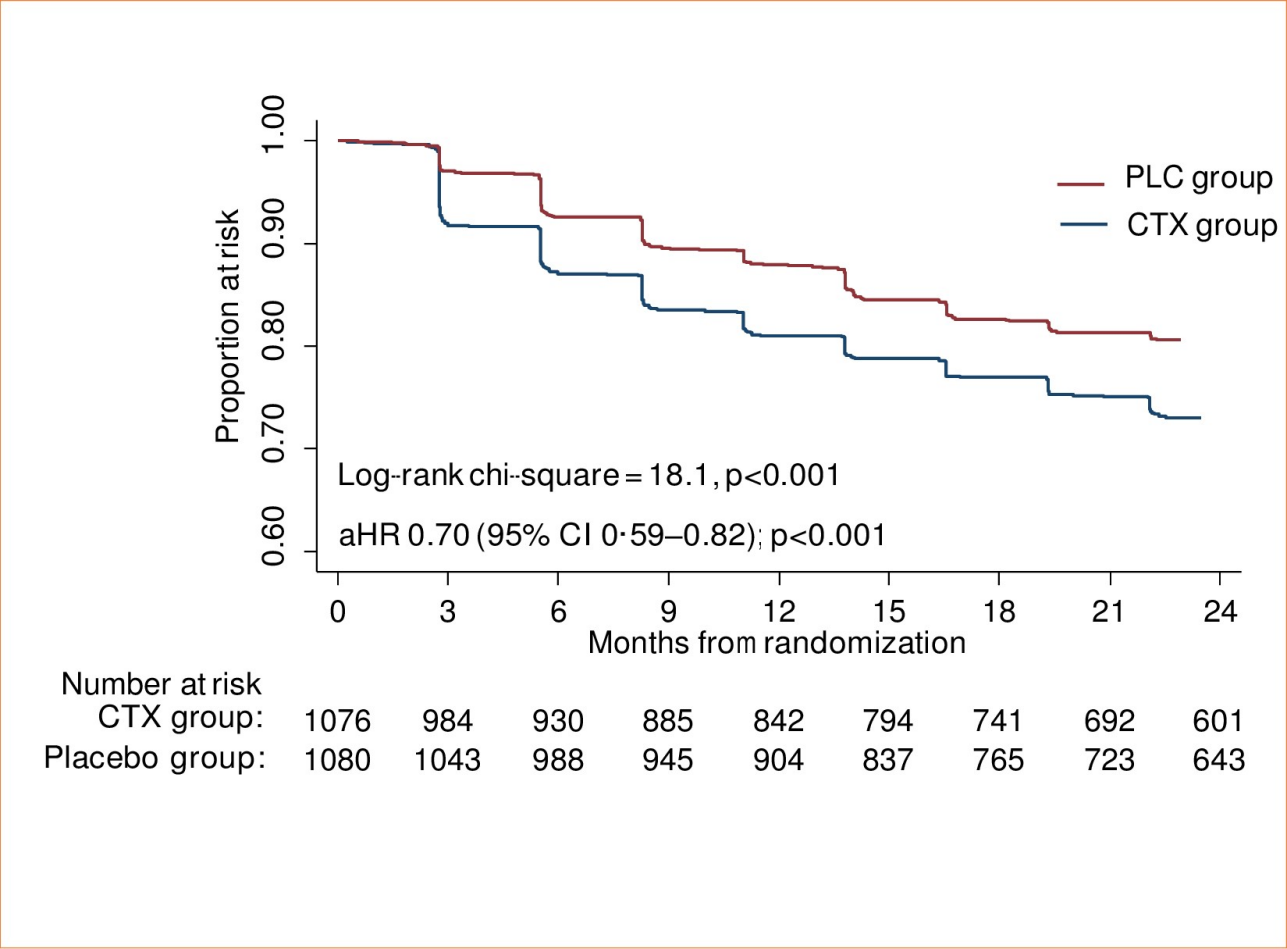
Time to first grade 3 / 4 haematological adverse event–ITT.

A total of 551 participants (318 CTX, 233 PLC) experienced a total of 1043 haematological adverse events (616 CTX, 427 PLC). An analysis of time to the first event showed a significant excess in the CTX group (log-rank χ2 = 18.08; P<0.0001) (Fig 3). This was confirmed by fitting a Cox proportional hazards regression model, adjusting for CD4 stratum and study site. The aHR for PLC *vs*. CTX was 0.70, (95% CI 0.59; 0.82). The excess was mainly due to grade 3 or 4 neutropenic events, with 28.2% of participants in the CTX group experiencing this at least once compared to 19.3% in the PLC group. The trial thus demonstrated the superiority of CPT discontinuation over continued CPT with respect to the primary haematological outcome.

### Secondary Outcomes

Results of secondary outcomes are shown in Table 3. There was no evidence of a difference in all-cause mortality between trial groups: 19 deaths in the CTX group (1.8%) and 18 in the PLC group (1.7%) (log-rank χ2 = 0.01; P = 0.91). Causes of death are listed in a table (S2 Table. Causes of death)

**Table 3:** Superiority analysis for primary safety outcome and secondary outcomes -ITT population.

Outcome	Placebo: participants with ≥1 event (%); (no. of events)	Placebo rate per 100 pyar (95% C.I. time to 1st event)	CTX: participants with ≥1 event (%); (no. of events)	CTX rate per 100 pyar (95% C.I. time to 1st event)	Stratified log-rank statistic (P-value)	Adjusted Hazard Ratio (95% C.I.)
**Primary safety outcome**						
Grade 3 / 4 haematological adverse event	233 (21.3%) 427 events	10.6 (9.4; 12.1)	318 (28.9%) 616 events	15.3 (13.7; 17.1)	18.08 (<0.0001)	0.7 (0.59; 0.82)
**Secondary outcomes**						
All-cause mortality	18 (1.7%)	0.73 (0.46; 1.16)	19 (1.8%)	0.76 (0.49; 1.19)	0.01 (0.91)	0.96 (0.50; 1.83)
All WHO stage 2, 3 or 4 events or death	97 (8.9%) 125 events	4.1 (3.4; 5.0)	77 (7.1%) 99 events	3.2 (2.6; 4.0)	2.53 (0.11)	1.28 (0.95; 1.72)
Confirmed clinical malaria	276 (25.3%) 350 events	13 (11.6; 14.7)	86 (7.9%) 103 events	3.6 (2.9; 4.4)	122.6 (<0.0001)	3.62 (2.84; 4.61)
Hospital admissions	93 (8.5%) 110 events	4 (3.3; 4.9)	53 (4.9%) 65 events	2.2 (1.7; 2.9)	12.36 (0.004)	1.82 (1.30; 2.50)
SAEs	102 (9.4%) 124 events	4.4 (3.6; 5.3)	67 (6.2%) 90 events	2.8 (2.2; 3.5)	8.44 (0.0037)	1.58 (1.16; 2.15)

More patients in the PLC group (97 participants, 8.9%, 125 events) experienced at least one WHO surveillance clinical stage 2,3 or 4 event or death than participants in the CTX group (77 participants, 7.1%, 99 events), a non-significant difference (log-rank χ2 = 2.53; P = 0.11 for time to first event). In total 362 (276 (25.3%) PLC, 86 (7.9%) CTX) participants experienced at least one episode of confirmed clinical malaria (P<0.0001). A detailed analysis of malaria events has been presented elsewhere.[19] More participants in the PLC group than in the CTX group were admitted to hospital at least once (93; 8.5% vs 53; 4.9%) (P = 0.0004). Reasons for hospitalisation are listed in a table (S3 Table. Reasons for hospitalisation). The proportion of participants experiencing at least one serious adverse event, comprising hospital admission, death or adverse pregnancy outcome (still births or miscarriages), was also higher in the PLC group (102; 9.4%) than in the CTX group (67; 6.2%) (P = 0.0037). A secondary endpoint was the rate of all grade 3 or grade 4 haematological adverse events allowing for multiple events per participant. An analysis allowing for the clustering of events within participants showed that participants on PLC were significantly less likely to experience grade 3 or 4 adverse events than those on CTX (aHR = 0.63; P<0.001). A comparison of mean changes in haematological parameters after 48 weeks, adjusting for the baseline value of the parameter, study site and actual number of days between the two measurements, is presented in Table 4. The mean CD4 count at week 48 was significantly higher in the PLC group (P<0.001), as was the total white blood cell count (P<0.001) and the neutrophil count (P<0.001). There was no significant difference between trial groups in haemoglobin levels at week 48 (P = 0.44), but some evidence that the platelet count at week 48 was higher in the CTX group (P = 0.08). Adherence to trial drug was very similar in the two group with 4.0% of patient-visits in the PLC group reporting taking less than 80% of trial drug compared to 3.7% in the CTX group (P = 0.58 using robust standard errors to allow for clustering within participants). The proportion of patient visits at which participants reported having missed a dose of ART was also similar (3.5% PLC *vs*. 3.3% CTX; P = 0.64) as was the proportion of patient visits at which participants reported not having slept under a mosquito net (5.2% PLC *vs*. 4.9% CTX; P = 0.42).

**Table 4:** Comparison of outcomes for continuous laboratory variables at week 48 between treatment groups.

Parameter	PLC group	CTX group	P-value
Baseline CD4 count mean (s.d.)	518.6 (198.8)	519.6 (192.4)	<0.001
Week 48 CD4 count mean (s.d.)	531.2 (200.9)	506.3 (188.8)	
Marginal mean at week 48 (95% c.i.)	498.2 (491.0; 505.5)	472.4 (466.0; 479.7)	
Baseline WBC count mean (s.d.)	4.64 (1.47)	4.61 (1.40)	<0.001
Week 48 WBC count mean (s.d.)	4.59 (1.40)	4.24 (1.37)	
Marginal mean at week 48 (95% c.i.)	4.39 (4.32; 4.46)	4.05 (3.99; 4.11)	
Baseline neutrophil count mean (s.d.)	1.82 (0.89)	1.78 (0.89)	<0.001
Week 48 neutrophil count mean (s.d.)	1.87 (0.95)	1.69 (0.90)	
Marginal mean at week 48 (95% c.i.)	1.67 (1.63; 1.72)	1.51 (1.47; 1.55)	
Baseline Hb count mean (s.d.)	13.36 (1.45)	13.31 (1.47)	0.33
Week 48 Hb count mean (s.d.)	13.36 (1.55)	13.26 (1.51)	
Marginal mean at week 48 (95% c.i.)	13.33 (13.27; 13.40)	13.29 (13.24; 13.34)	
Baseline platelets count mean (s.d.)	261.5 (68.8)	262.5 (72.9)	0.18
Week 48 platelets count mean (s.d.)	258.2 (74.7)	263.3 (73.2)	
Marginal mean at week 48 (95% c.i.)	253.6 (250.1; 257.0)	256.9 (253.6; 260.2)	

A sensitivity analysis was performed by analysing as an end point all CTX-preventable events or all-cause mortality (i.e. including the 31 deaths deemed not to be CTX-preventable). The aHR comparing placebo *vs*. CTX was 1.37 (upper limit of the one-sided 95% CI 1.85). This upper 95% confidence limit was still beyond the non-inferiority cut-off of 1.25. In a further sensitivity analysis in which we excluded 163 participants who admitted to having taken open label CTX at least once (80 CTX; 83 PLC) the aHR was 1.51 (upper limit of the one-sided 95% CI2.13), and this too did not achieve non-inferiority.

## Discussion

Our study evaluated the benefits and risks of discontinuing CPT in HIV infected adults who are stable on ART in the Uganda. We found that discontinuing CPT significantly increased the risk of pre-defined CTX-preventable infections, malaria and related hospital admissions. On the other hand, discontinuing CPT substantially reduced the risk of serious haematologic adverse events, in particular neutropenia, and did not lead to an increase in mortality.

We did not demonstrate non-inferiority of discontinuing CPT in regard to preventing severe bacterial infections and malaria and in this respect, our results are in agreement with earlier open-label studies.[15,16] Cotrimoxazole is a broad spectrum combination antimicrobial (trimethoprim/sulfamethoxazole) that is effective against many common bacterial and protozoal pathogens. Therefore, the effects of continued CPT may have been expected and a similar effect would probably be observed among HIV negative individuals [26]. However, primary prophylaxis against bacterial infections with broad spectrum antimicrobials is not generally recommended for HIV-negative individuals, primarily to guard against widespread antimicrobial drug resistance; a concern that has previously been raised by others.[27] Further, cotrimoxazole is not the drug of choice for malaria prophylaxis,[28] although prevention of malaria in HIV infected patients is an accepted benefit of CPT [17]. It is also important to note that malaria is still likely to occur among patients on CTX as we also observed a non-negligible proportion of patients on CTX who still got malaria. This implies that other malaria preventive strategies should complement CTX prophylaxis. In the context of HIV infection therefore, CPT offers combined prevention of co-morbid bacterial and protozoal infections as an adjunct to ART in patients with moderate immune suppression.[29]

We demonstrated that patients who continue CPT have an increased risk of haematological abnormalities, mainly neutropenia, as well as a lower CD4 count increase under effective ART compared to those who stopped CPT. Neutropenia is likely to be the result of cotrimoxazole induced bone marrow suppression, owing to the known antifolate effect of the sulphur moiety contained in the drug.[6,8] This mechanism may also explain the attenuated increase in CD4 counts that we observed in the cotrimoxazole group (Table 4). This finding supplements previous studies on CPT among patients on ART which did not report comparisons of the haematological adverse drug effects of cotrimoxazole.[12,15]

The major strengths of our study were the large sample, the double-blind placebo-controlled randomized design, long follow up time and adjudication of clinical outcomes by an independent ERC. That we evaluated haematological adverse events as a co- primary endpoint is an additional strength.

Our study had some limitations. The observed rate of CTX-preventable events was much lower than that assumed in the sample size calculations. This could have reduced the power of the trial to show non-inferiority with respect to CTX-preventable events. However in this case we were able to show that continuing CPT was in fact superior to stopping CPT with respect to CTX-preventable events, so the study was in fact adequately powered to demonstrate superiority. The PP population was defined by self-reported adherence to study drug. Adherence to study drug was generally good but not perfect, nor was this the case for the adherence to ART or bed net use. However, adherence was similar between trial groups, and if at all a lack of adherence would have diluted the measurable effects of the intervention (use of placebo rather than CTX). This may have resulted in misclassification, inflating the PP population. The adjudication of CTX-preventable effects may also have led to misclassification, diluting measurable differences between trial groups. This risk was small as clinical observations were carefully recorded and the ERC comprised highly experienced clinicians. The exit interviews[20] showed some evidence of open label drug being taken by participants, although results from the interviews do not suggest that trial results would have been seriously compromised. Finally, response to ART was determined by CD4 count, and not viral load. As virological failure on ART may precede CD4 count decline, some patients could have been at a higher risk of CTX-preventable events than expected. Due to the randomised design, however, this would not have affected the comparability of trial groups.

The effect of CPT discontinuation was greater in the higher CD4 count stratum. This finding is surprising and we have no clear explanation for it. However, as a point of further research, one could speculate that an increased expression of CTX-preventable events occurs at higher CD4 counts. Our data show that the number needed to treat with CPT for one year in order to prevent one severe bacterial infection was 113. While we showed that continuing CPT reduced the risk of severe bacterial infections, from a public health point of view this might not be worthwhile, particularly in settings where there is a low level of such infections, in patients that have been immunologically reconstituted by ART and / or have adequate management of these infections. In this respect, our results have important implications for HIV treatment policy in those countries that still recommend continued CPT alongside ART. A cost effectiveness analysis based on data from the COSTOP trial is currently ongoing and will provide added information on the cost benefits of CPT during ART.

In conclusion, continued CPT is beneficial for ART stable HIV infected patients who are at high risk for severe bacterial infections or malaria. This benefit needs to be weighed against the risk of haematologic toxicity, and patients eligible for CPT should have access to CD4 and complete blood count haematological side effects monitoring.

## Supporting information

S1 Checklist CONSORT 2010 checklist. (DOC)

S1 Appendix Cotrimoxazole preventable events. (DOCX)

S1 Table Non-fatal cotrimoxazole preventable events by treatment group. (DOCX)

S2 Table Causes of death. (DOCX)

S3 Table Reasons for hospitalization. (DOCX)

S1 File COSTOP trial design manuscript. (PDF)

S2 File UVRI REC COSTOP trial initial approval. (PDF)

S3 File COSTOP clinical trial protocol. (PDF)
