# Supplementary material for: Discontinuing cotrimoxazole preventive therapy in HIV-infected adults who are stable on antiretroviral treatment in Uganda (COSTOP): A randomised placebo controlled trial
Source: PLoS One. 2018 Dec 31;13(12):e0206907. doi: 10.1371/journal.pone.0206907 (PMC6312229; doi:10.1371/journal.pone.0206907)
Supplement: S1 Appendix — (DOCX) [file pone.0206907.s002.docx]

**S1 Appendix. Cotrimoxazole preventable events**

**WHO clinical stage 4**

Pneumocystis pneumonia

Recurrent severe bacterial pneumonia

Central nervous system toxoplasmosis

Chronic isosporiasis

Recurrent non typhoidal salmonella bacteraemia

**WHO clinical stage 3**

Unexplained severe weight loss (>10% of presumed or measured body weight)

Unexplained chronic diarrhoea for longer than one month

Unexplained persistent fever (above 37.6°C intermittent or constant, for longer than one month)

Severe bacterial infections (such as pneumonia, empyema, pyomyositis, bone or joint infection, meningitis or bacteraemia)

Acute necrotizing ulcerative stomatitis, gingivitis or periodontitis

Unexplained anaemia (<8 g/dl), neutropaenia (<0.5 × 10⁹ per litre) or chronic thrombocytopaenia (<50 × 10⁹ per litre)

**WHO clinical stage 2**

Moderate unexplained weight loss (<10% of presumed or measured body weight)

Recurrent respiratory tract infections; sinusitis, tonsillitis, otitis media and pharyngitis

**Source**: World Health Organisation. WHO case definitions of HIV for surveillance and revised clinical staging and immunological classification of HIV-related disease in adults and children. 7 August 2006.
