# Supplementary material for: Discontinuing cotrimoxazole preventive therapy in HIV-infected adults who are stable on antiretroviral treatment in Uganda (COSTOP): A randomised placebo controlled trial
Source: PLoS One. 2018 Dec 31;13(12):e0206907. doi: 10.1371/journal.pone.0206907 (PMC6312229; doi:10.1371/journal.pone.0206907)
Supplement: S1 Table — (DOCX) [file pone.0206907.s003.docx]

**S1 Table. Non-fatal cotrimoxazole preventable events by treatment group**

| **Description of event** | **CTX group** | **Placebo group** | **Total** |
| --- | --- | --- | --- |
| Recurrent bacterial upper respiratory tract infections | 5 | 4 | 9 |
| Sinusitis | 1 | 1 | 2 |
| Cellulitis | 0 | 3 | 3 |
| Acute appendicitis | 0 | 1 | 1 |
| Otitis media | 2 | 1 | 3 |
| Atypical pneumonia | 0 | 1 | 1 |
| Bartholin’s abscess | 1 | 0 | 1 |
| Bacterial bronchopneumonia | 20 | 33 | 53 |
| Epididymorchitis | 1 | 0 | 1 |
| Infected uterine fibroids | 0 | 1 | 1 |
| Infective endocarditis | 0 | 1 | 1 |
| Lobar pneumonia | 1 | 3 | 4 |
| Septic skin lesions | 0 | 2 | 2 |
| Skin abscess | 1 | 1 | 2 |
| Pelvic abscess | 1 | 0 | 1 |
| Pelvic inflammatory disease | 3 | 2 | 5 |
| Perirectal abscess | 0 | 1 | 1 |
| Pharyngitis | 1 | 0 | 1 |
| Puerperal sepsis | 2 | 1 | 3 |
| Pyelonephritis | 1 | 0 | 1 |
| Pyomyositis | 0 | 2 | 2 |
| Septic arthritis | 0 | 2 | 2 |
| Septicaemia | 3 | 0 | 3 |
| Submandibular abscess | 0 | 1 | 1 |
| Urinary tract infection | 1 | 4 | 5 |
| Tracheobronchitis | 1 | 0 | 1 |
| Chronic unexplained diarrhoea | 1 | 1 | 2 |
| **Total** | **46** | **66** | **112** |
