# Supplementary material for: Discontinuing cotrimoxazole preventive therapy in HIV-infected adults who are stable on antiretroviral treatment in Uganda (COSTOP): A randomised placebo controlled trial
Source: PLoS One. 2018 Dec 31;13(12):e0206907. doi: 10.1371/journal.pone.0206907 (PMC6312229; doi:10.1371/journal.pone.0206907)
Supplement: S2 Table — (DOCX) [file pone.0206907.s004.docx]

**S2 Table. Causes of death**

Deaths as a result of a “CTX-preventable event” according to ERC adjudication

| **Description of event** | **Treatment group** | |
| --- | --- | --- |
|  | **CTX** | **Placebo** |
| Diarrhoea of unknown cause |  | 1 |
| Kaposi’s sarcoma with severe sepsis |  | 1 |
| Klebsiella pneumonia |  | 1 |
| Malaria with quinine toxicity | 1 |  |
| Pyogenic meningitis | 1 |  |
| Septicaemic shock |  | 1 |
| **Total** | **2** | **4** |

Death **not** a result of “CTX-preventable events” according to ERC adjudication

| **Description of event** | **Treatment group** | |
| --- | --- | --- |
|  | **CTX** | **Placebo** |
| Alcohol intoxication | 1 | 0 |
| Cancer of the cervix | 0 | 3 |
| Carcinoma of the breast | 1 | 1 |
| Carcinoma of the oesophagus | 2 | 0 |
| Congestive cardiac failure – rheumatic heart disease | 0 | 1 |
| Congestive cardiac failure – Cor pulmonale | 1 | 0 |
| Dilated cardiomyopathy | 1 | 1 |
| Encephalitis – undetermined cause | 0 | 1 |
| Lactic acidosis | 1 | 0 |
| Liver failure – suspected hepatocellular carcinoma | 0 | 1 |
| Liver failure – undiagnosed chronic liver disease | 1 | 0 |
| Multiple crash injuries – road traffic accident | 1 | 0 |
| Postpartum haemorrhage | 1 | 1 |
| Pulmonary embolism – secondary to deep venous thrombosis | 1 | 0 |
| Pulmonary tuberculosis | 2 | 2 |
| Severe anaemia – AZT related, with congestive cardiac failure | 1 | 0 |
| Severe anaemia – undiagnosed haematologic malignancy | 0 | 1 |
| Severe iron deficiency anaemia – undiagnosed malignancy | 0 | 1 |
| Status epilepticus | 1 | 0 |
| Unknown | 2 | 1 |
| **Total** | **17** | **14** |
