# Supplementary material for: Discontinuing cotrimoxazole preventive therapy in HIV-infected adults who are stable on antiretroviral treatment in Uganda (COSTOP): A randomised placebo controlled trial
Source: PLoS One. 2018 Dec 31;13(12):e0206907. doi: 10.1371/journal.pone.0206907 (PMC6312229; doi:10.1371/journal.pone.0206907)
Supplement: S3 Table — (DOCX) [file pone.0206907.s005.docx]

**S3 Table. Reasons for hospitalisation**

| **Condition leading to hospitalisation** | **Total** | **CTX group** | **Placebo group** |  | **Condition leading to hospitalisation** | **Total** | **CTX group** | **Placebo group** |
| --- | --- | --- | --- | --- | --- | --- | --- | --- |
| **Anaemia** | **12** | **4** | **8** |  | **Surgical admissions** | **26** | **9** | **17** |
| Haemolytic (malaria) |  |  | 1 |  | Acute urinary retention – urethral stricture |  | 1 |  |
| Haemorrhagic |  | 1 | 1 |  | Breast carcinoma – diagnostic biopsy |  |  | 1 |
| Macrocytic - undiagnosed |  |  | 1 |  | Cervical carcinoma – exploratory laparatomy |  |  | 1 |
| Megaloblastic |  |  | 2 |  | Cervical polyp - excision |  |  | 1 |
| Microcytic - undiagnosed |  |  | 1 |  | Excision biopsy (Kaposi’s sarcoma ulcer) |  |  | 1 |
| Secondary to chronic illness |  | 3 | 1 |  | Ectopic pregnancy |  | 1 | 1 |
| Pancytopenia - undiagnosed |  |  | 1 |  | Hernia repair |  | 1 | 1 |
| **Cancer related (non-surgical)** | **3** | **2** | **1** |  | Hysterectomy |  | 1 | 2 |
| Chemotherapy, breast carcinoma |  | 1 |  |  | Myomectomy – uterine fibroids |  | 1 | 1 |
| Chemotherapy, cervical carcinoma |  |  | 1 |  | Pelvic abscess |  | 1 |  |
| Haemoptysis, breast carcinoma |  | 1 |  |  | Subacute intestinal obstruction |  |  | 1 |
| **Cardiovascular disease** | **9** | **5** | **4** |  | Thoracocentesis – empyema thoracis |  |  | 1 |
| Heart failure – dilated cardiomyopathy |  | 3 | 2 |  | Thyroidectomy – multinodular goitre |  | 1 |  |
| Hypertension - uncontrolled |  | 1 | 2 |  | Trauma - accidental |  | 2 | 5 |
| Arrhythmias |  | 1 |  |  | Varicectomy – testicular veins |  |  | 1 |
| **Infectious illness** | **68** | **18** | **50** |  | **Obstetric admissions** | **7** | **1** | **6** |
| Bronchiectasis |  |  | 1 |  | Elective caesarean section |  |  | 3 |
| Bronchopneumonia |  | 1 | 5 |  | Intrapartum haemorrhage |  |  | 1 |
| Cellulitis |  |  | 1 |  | Obstructed labour |  | 1 |  |
| Cholecystitis |  |  | 1 |  | Pre-term labour |  |  | 2 |
| Lobar pneumonia |  |  | 1 |  | **Adverse pregnancy outcomes** | **10** | **6** | **4** |
| Pelvic inflammatory disease |  | 1 | 1 |  | Incomplete/complete spontaneous abortion |  | 5 | 2 |
| Puerperal sepsis |  | 1 | 1 |  | Missed abortion |  |  | 1 |
| Infected genital warts |  |  | 1 |  | Molar pregnancy |  |  | 1 |
| Septic arthritis |  |  | 1 |  | Stillbirth |  | 1 |  |
| Urinary tract infection |  | 1 | 1 |  | **Other reasons for hospitalisation** | **19** | **11** | **8** |
| Pulmonary tuberculosis |  | 1 | 2 |  | Acute abdominal pain - cholelithiasis |  |  | 1 |
| Oro-oesophageal candidiasis |  |  | 1 |  | Acute abdominal pain - undiagnosed |  | 2 | 2 |
| Malaria |  | 13 | 33 |  | Acute bronchial asthma |  | 1 | 2 |
|  | | | |  | Acute diarrhoea - undiagnosed |  | 1 |  |
|  |  |  |  |  | Acute febrile illness - undiagnosed |  | 1 | 2 |
|  |  |  |  |  | Acute gastroenteritis - undiagnosed |  | 1 |  |
|  |  |  |  |  | Anxiety |  | 2 |  |
|  |  |  |  |  | Peptic ulcer disease |  | 2 |  |
|  |  |  |  |  | Tension headache |  | 1 | 1 |
|  |  |  |  |  | **Total number of hospitalisations** | **154** | **56** | **98** |
