# Supplementary material for: Discontinuing cotrimoxazole preventive therapy in HIV-infected adults who are stable on antiretroviral treatment in Uganda (COSTOP): A randomised placebo controlled trial
Source: PLoS One. 2018 Dec 31;13(12):e0206907. doi: 10.1371/journal.pone.0206907 (PMC6312229; doi:10.1371/journal.pone.0206907)
Supplement: S2 File — (PDF) [file pone.0206907.s007.pdf]

Tel: (256) 41- 321962 (Direct)
(256) 41- 320385/6 (General)
Fax: (256) 41- 320483
Email: directoruvri@ug.cdc.gov

FOR GOD AND MY COUNTRY
THE REPUBLIC OF UGANDA

UGANDA VIRUS RESEARCH
INSTITUTE (UVRI)
P.O.Box 49 ENTEBBE (U)

Our Ref : GC/127/10/07/02
Your Ref:

1st July 2010

Prof. Heiner Grosskurth, Drs. Paula Munderi, Anatoli Kamali, et-al,

RE: UVRI SEC review of an amendment to the protocol titled: "**Safety of Discontinuing Cotrimoxaxole Prophylaxis among HIV Infected Adults in Uganda (COSTOP)**"

Thank you for your response to the queries addressed to you during the SEC meeting of 17th June 2010.

This is to inform you that your responses dated 30th June 2010 were reviewed and met the requirements of the UVRI Science and Ethics Committee.

UVRI SEC approval has been given for you to continue with your research. Annual progress report and request for extension should be submitted to UVRI SEC before the expiry of one year after initial date of approval i.e. by 10th March 2011.

You can now continue with your study after registration of your amendment with the Uganda National Council for Science and Technology (UNCST).

Wish you the best of luck.

Yours sincerely,

Mr. Tom Lutalo
Chair, UVRI SEC

C.C Director, UVRI
Secretary, UVRI SEC
