# Supplementary material for: Discontinuing cotrimoxazole preventive therapy in HIV-infected adults who are stable on antiretroviral treatment in Uganda (COSTOP): A randomised placebo controlled trial
Source: PLoS One. 2018 Dec 31;13(12):e0206907. doi: 10.1371/journal.pone.0206907 (PMC6312229; doi:10.1371/journal.pone.0206907)
Supplement: S3 File — (PDF) [file pone.0206907.s008.pdf]

# COSTOP

ACRONYM: COSTOP

Full Title of trial:

Safety of discontinuing Cotrimoxazole prophylaxis among HIV infected adults on ART in Uganda. A randomised controlled trial.

ISRCTN: **ISRCTN44723643**

Protocol number 5.0 Protocol date: 30th June 2010

................................ August 1st 2011

Authorised signature Date

Name Dr Edward Katongole-Mbidde

(Chair of TSC for final protocols and amended final protocols)

# GENERAL INFORMATION

This document describes the COSTOP trial and provides information about procedures for entering patients into it. The protocol should not be used as an aide-memoire or guide for the treatment of other patients; every care was taken in its drafting, but corrections or amendments may be necessary. These will be circulated to the registered investigators in the trial. Clinical problems relating to this trial should be referred to the relevant Chief Investigator.

**Compliance:** The trial will be conducted in compliance with the present protocol and MRC GCP, Data protection Act (DPA number: Z5886415). The trial will adhere to Uganda Virus Research Institute (UVRI) Science and Ethics Committee and Uganda National Council of Science and Technology (UNCST) research governance and to the regulatory requirements of Uganda National Drug Regulatory Authority (NDA).

**Principal Investigators (in bold), Co-Principal Investigators & Investigators responsible for conducting trial**


**Professor Heiner Grosskurth** ***heiner.grosskurth@mrcuganda.org***
Dr Paula Munderi *paula.munderi@mrcuganda.org*
Dr Anatoli Kamali *anatoli.kamali@mrcuganda.org*
Dr Ronald Kasirye *ronnie.kasirye@mrcuganda.org*
*MRC/UVRI Uganda Research Unit on AIDS*
*Uganda Virus Research Institute*
*P O Box 49, Entebbe Uganda*

Professor Andrew Nunn *ajn@ctu.mrc.ac.uk*
*MRC Clinical Trials Unit*
*222 Euston Road*
*London NW1 2DA*


**Trial Statistician**


Dr Jonathan Levin *jonathan.levin@mrcuganda.org*
*MRC/UVRI Uganda Research Unit on AIDS*
*Uganda Virus Research Institute*
*P O Box 49, Entebbe Uganda*


**Collaborators:**
TASO Entebbe Center
TASO Masaka Center

**Trial Monitor:** TBA
The trial will be conducted in compliance with the protocol, GCP and regulatory requirements

**Clinical laboratories:** MRC Laboratories Entebbe ; MRC Laboratories Masaka

**Trial funders:** MRC (UK)

**Important Contacts:**

**SAE NOTIFICATION**

Within one working day of becoming aware of an SAE, please email a completed SAE form to the Study coordinator on <ronnie.kasirye@mrcuganda.org>

# TABLE OF CONTENTS


**1. Summary ........................................ 8**

1.1 Summary of trial design ........................................ 8
1.2 Flow diagram ........................................ 9

**2. Background ........................................ 10**

2.1 Introduction ........................................ 10
2.2 Rationale and objectives ........................................ 12

**3. Selection of Centres/Clinicians ........................................ 13**

**4. Selection of Patients ........................................ 13**

4.1 Patient inclusion criteria ........................................ 13
4.2 Patient exclusion criteria ........................................ 13
4.3 Screening procedures and pre-randomisation investigations ........................................ 14

**5. Randomisation & Enrolment procedure .... 14**

5.1 Randomisation practicalities ........................................ 14
5.2 Randomisation codes and unblinding ........................................ 15
5.3 Co-enrolment guidelines ........................................ 15

**6. Treatment of Patients ........................................ 15**

6.1 Trial treatment ........................................ 15
6.2 Trial product(s) ........................................ 15
6.3 Dispensing ........................................ 15
6.4 Modification of trial treatment ........................................ 16
6.5 Measures of adherence ........................................ 16
6.6 Non-trial treatment ........................................ 16

**7. Assessments and follow-up ........................................ 16**

7.1 Schedule for follow-up / Flow Chart ........................................ 16
7.2 Procedures for assessing efficacy ........................................ 17
7.3 Procedures for assessing safety ........................................ 18
7.4 Loss to follow-up ........................................ 18
7.5 Trial closure ........................................ 18

**8. Withdrawal of patients ........................................ 18**

8.1 Withdrawal from trial intervention ........................................ 18
8.2 Patient transfers ........................................ 19
8.3 Withdrawal from the trial completely ........................................ 19

**9. Statistical Considerations ........................................ 19**

9.1 Method of Randomisation ........................................ 19
9.2 Outcome Measures ........................................ 19
9.3 Sample Size ........................................ 20
9.4 Interim Monitoring and Analyses ........................................ 20
9.5 Analysis Plan (brief) ........................................ 21

**10. Trial Monitoring ........................................ 22**

10.1 Monitoring in the Data Section at MRC/UVRI Entebbe ........................................ 22
10.2 Clinical Site Monitoring ........................................ 22

11. Safety Reporting ........................................ 23
11.1 Definitions ........................................ 23
11.2 Institution/Investigator Responsibilities ........................................ 24
11.3 MRC / UVRI Responsibilities ........................................ 26
12. Ethical Considerations and Approval ......... 28
12.1 Ethical considerations ........................................ 28
12.2 Ethical approval ........................................ 28
13. Indemnity ........................................ 28
14. Trial Committees ........................................ 29
14.1 Trial Management Group (TMG) ........................................ 29
14.2 Trial Steering Committee (TSC) ........................................ 29
14.3 Independent Data Monitoring Committee (IDMC) ........................................ 29
14.4 Endpoint Review Committee (ERC) ........................................ 29
15. Publication ........................................ 29
16. Protocol Amendments ........................................ 30
17. References ........................................ 33
18. Appendices ........................................ 34

## APPENDICES

APPENDIX 1: PATIENT INFORMATION SHEET ........................................ 35
APPENDIX 2: CONSENT FORM ........................................ 38
APPENDIX 3: TOXICITY TABLE ........................................ 40
CLINICAL EVENTS CRITERIA ........................................ 52
TOXICITY MANAGEMENT TABLES ........................................ 59
APPENDIX N: CASE REPORT FORMS ........................................ 58

## ABBREVIATIONS AND GLOSSARY

| | |
|---|---|
| AE | Adverse event |
| AR | Adverse reaction |
| ART | Antiretroviral Therapy |
| CF | Consent form |
| CRF | Case Report Form |
| CTX | Cotrimoxazole |
| ERC | Endpoint Review Committee |
| IDMC | Independent Data Monitoring Committee |
| ISRCTN | International standard randomised controlled trial number |
| MRC CTU | MRC Clinical Trial Unit, London |
| MRC | Medical Research Council |
| PI | Principal Investigator |
| PIS | Patient information Sheet |
| SAE | Serious adverse event |
| SAR | Serious adverse reaction |
| SOP | Standard operating procedures |
| SUSAR | Suspected unexpected serious adverse reaction |
| TMG | Trial Management Group |
| TSC | Trial Steering Committee |
| UAR | Unexpected adverse reaction |

# 1. SUMMARY

## 1.1 Summary of trial design

The COSTOP protocol describes a randomised double blind placebo controlled non-inferiority trial to evaluate whether long-term primary and secondary prophylaxis with cotrimoxazole can be safely discontinued among Ugandan adults on antiretroviral therapy who have achieved sustained immune restoration (measured as a confirmed increase in CD4 count to 250 or more cells/mm$^3$)

Eligible patients will be HIV +ve adults aged 18 years and above who are stable on ART and have confirmed sustained CD4 restoration to 250 cells/mm3 and above with no contraindication to being randomised to placebo or control groups and who are able to attend designated study clinics.

Patients who are eligible will be randomised 1:1 to the experimental or control group:

*Experimental group:* one oral tablet of placebo CTX daily

*Standard/Control group:* one oral tablet of 960 mg of CTX daily

All patients will continue to take their ART

There are two co-primary outcome measures, one for efficacy and one for safety.

- The efficacy outcome measure is the time to the occurrence of the first clinical event (pre-defined CTX-preventable opportunistic clinical event or death).
- The safety outcome measure is the time to the occurrence of the first grade 3 or 4 haematological adverse event.

Secondary outcome measures will be:

1. Incidence of all CTX preventable events
2. All cause mortality
3. Incidence of all clinical events and related events requiring hospitalisation
4. Incidence of all confirmed malaria episodes* asymptomatic and symptomatic
5. Severity and outcome of all confirmed malaria episodes* asymptomatic and symptomatic.
6. Incidence of grade 3 or grade 4 adverse events.
7. Mean change in CD4 count after 12 months on the trial.
8. Mean change in haematologic indices after 12 months on the trial.
9. Serious Adverse Events (SAEs)-according to ICH/GCP definitions.
10. Adherence to use of ART, trial drug and insecticide-treated mosquito nets.

* confirmed by positive parasitaemia on a blood slide.

COSTOP is a three year trial, recruitment into the trial will take place over 18 months and patients will be followed for a minimum of 18 months and a maximum of 36 months.

The study is funded by the MRC (UK) and is to be conducted by the MRC/UVRI Uganda Research Unit on AIDS.

## 1.2 Flow diagram

**Figure 1: Trial entry, randomisation and treatment**

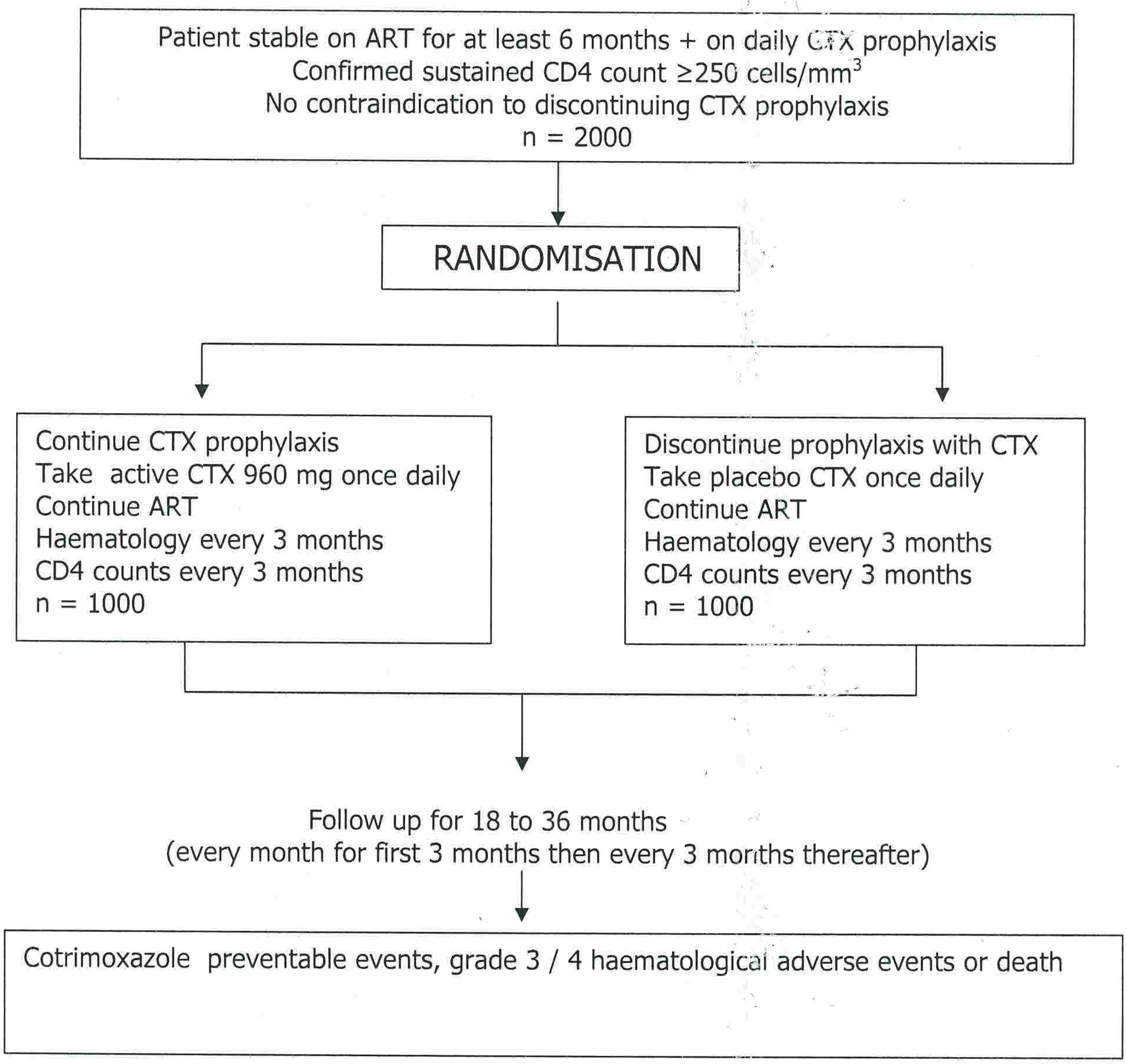

# 2. BACKGROUND

## 2.1 Introduction

In Uganda, cotrimoxazole (CTX) prophylaxis for life for all HIV-infected persons was adopted as policy in 2005[1]. This policy is based on studies in Africa [2-8] which demonstrated marked reductions in HIV-related morbidity and mortality and on recommendations from WHO.[9]
All these studies were done in patients not yet on antiretroviral therapy (ART). The benefit of concurrent prophylaxis with CTX has been attributed to reduction in rates of CTX preventable infectious complications of HIV.[3,6].

Once HIV infected patients in Africa have commenced ART, the benefits of continuing to provide additional prophylactic treatment with CTX indefinitely are not known. Indeed there are several potential disadvantages of continuing to prescribe CTX alongside ART : most importantly, co-toxicity with ARVs, such as haemotoxicity when CTX is combined with zidovudine[10]; increased pill burden leading to possible pill fatigue in the long term and diminished adherence to HIV treatment; as well as increased overall costs of HIV care.

With the advent of widespread ART in Africa, policy guidance on CTX prophylaxis needs to be re-evaluated. Current policy guidelines highlight the lack of reliable information on when to discontinue prophylaxis with CTX in resource-limited settings[1,9] and recommend further research. Studies are required now, in patients receiving CTX and ART concurrently, to evaluate the usefulness and safety of continued prophylaxis with CTX once immune function has been restored by ART. Concern about the potential for additive haemotoxicity is amplified by recent ART guidelines revision in Uganda which now recommend that the preferred standard first line ART regime be Zidovudine/ Lamivudine/Nevirapine .[11]

### 2.1.1 Relevant studies/trials

In resource-rich settings, randomised controlled trials have demonstrated that once HIV-infected persons are on ART and have attained CD4 counts of 200 or more cells/mm$^3$ for at least 3 months, prophylaxis with CTX can be discontinued safely[12] without increasing their risk of major cotrimoxazole preventable opportunistic infections.

Lowrance D and colleagues in Malawi have recently reported a 40.7% reduction in risk of mortality within the first six months of initiating ART associated with attending an ART clinic that also routinely offered CTX prophylaxis. This was a non-randomised retrospective cohort study with a short period of follow up.[13] It should be noted that:

a) Although the authors investigated the 6-month mortality of patients initiating ART no data on immune function of the patients are reported. It is quite likely that a significant proportion of patients at this early stage of treatment would have had CD4 counts below 250 cells/mm$^3$. Thus the protective effect of CTX that they found is insufficient to prove that primary prophylaxis should continue beyond CD4 recovery to 250 cells/mm$^3$.

b) As the authors point out this was a non-randomised comparison of patients in 5 ART sites that provided CTX, with patients in 6 ART sites that did not provide CTX chosen as being "comparable". This was a major weakness of this study as the health care offered in the 5 sites providing CTX prophylaxis might have been of a higher standard than that in the 6 sites that did not provide CTX (ability to provide CTX prophylaxis may in itself be evidence for providing "better care").

Furthermore there is no mention in the Malawi paper that analysis took into account the clustering of patients within these 11 sites – hence the statistical significance is likely to have been inflated. This study therefore does not answer the current research question.

More recently, in a home based care setting in rural Eastern Uganda, investigators of the Centre for Disease Control (CDC) randomised 836 patients who had been on ART for a median of 3.7 years; to continue or discontinue CTX prophylaxis (at median CD4 counts of 476 and 505 cells / mm3 respectively). This randomisation was stopped prematurely after only 116 days of follow up by the DSMC, following occurrence of significantly higher rates of asymptomatic and symptomatic malaria in the group which stopped CTX. (RR = 28, 95%CI 6 -105, p<0.0001). [14] There was no difference in mortality. ***[Campbell James; personal communication].*** This study did not answer the question whether CTX can prevent or reduce HIV-related morbidity, although CTX had an impact on episodes of Malaria parasitaemia.

Further to this, a non-randomised observational analysis evaluated the benefits of using CTX after ART initiation in the DART study, where CTX was to be prescribed along with ART at the discretion of the treating clinicians. Of the 3179 participants in DART, 62% were receiving CTX at ART initiation, 28% commenced while on ART, and 10% never received it during follow-up (median, 4.9 years). Overall, CTX co-treatment reduced risk of mortality by 35% (odds ratio, 0.65; 95% CI, 0.50–0.85). This reduction was greatest in the first 12 weeks of concurrent use with ART (OR, 0.41; 95% CI, 0.27–0.65) and remained statistically significant up to 72 weeks after starting ART, (OR, 0.56; 95% CI, 0.37–0.86). Thereafter, there was no additional reduction in mortality observed. CTX prophylaxis also reduced malaria risk by 26% (OR, 0.74; 95% CI, 0.63–0.88), and this benefit was maintained throughout follow-up. Paradoxically, no beneficial effect was observed on occurrence of WHO clinical staging events, change in CD4 count or body mass index.

CTX is not standard recommended prophylaxis against malaria in Uganda as more appropriate interventions are available in practice.[15] Provided complete information is availed to potential study participants and as long as appropriate malaria control guidelines are adhered to, it remains important that a study with all cause morbidity as an end point be carried out, to provide answers to the outstanding questions.

In December 2008, a consultative workshop attended by researchers and ethical and regulatory bodies was convened by the Uganda National Council for Science and Technology (UNCST) to discuss the scientific rationale and ethical appropriateness of continuing CTX cessation studies in Uganda in light of the CDC data cited above. This consultation concluded that there is genuine scientific/clinical equipoise regarding whether and when to stop use of CTX as prophylaxis against opportunistic infections in people living with HIV who are also on ART. [**UNCST Consultation Report – Annex I**]

A similar randomised double-blind clinical trial has recently received approval for funding by the AIDS Clinical Trials Group (ACTG) and is to be carried out in Moshi, Tanzania and other areas of *low* Malaria transmission. In this study - *ACTG 5270*, 1660 clinically stable patients will be recruited and followed up for 3.5 years. Cotrimoxazole-preventable morbidity events will be the primary outcome and analyses will be stratified by entry CD4 counts at 200-350, 350-500 and >500 cells/mm$^3$. ***[John Barlett, personal communication].*** Results from this study and ours would be complementary, providing required evidence from contrasting settings.

### 2.1.2 Population

The trial will be carried out at 2 MRC Uganda study sites (Entebbe and Masaka). In each site patients who have been stabilized on ART with a CD4 count over 250 cells/mm$^3$ will be recruited provided they satisfy the inclusion and exclusion criteria and give their consent.

### 2.1.3 Investigational product/ intervention(s)

Experimental group: Patients with CD4 count 250 or more cells/mm$^3$ discontinue prophylaxis with CTX , receive CTX placebo and continue taking ART.

Standard/Control group: Patients with CD4 count 250 or more cells/mm$^3$ continue prophylaxis with CTX , receive active CTX and continue taking ART.

## 2.2 Rationale and objectives

Principle Question: Can long-term primary and secondary prophylaxis with cotrimoxazole be safely discontinued among African adults on antiretroviral therapy who have achieved sustained immune restoration (measured as a confirmed increase in CD4 count to 250 or more cells/mm$^3$)?

The objective of the study is to assess whether, in patients with CD4 count 250 or more cells/mm$^3$, the intervention regimen of discontinuation of CTX prophylaxis is
(a) not inferior to the control regimen in which prophylaxis with CTX is continued and
(b) superior with respect to the incidence of haematological adverse events..

i.e. The hypothesis of the study is that stopping CTX will not lead to an excess of clinical events (predefined CTX preventable clinical events or death) but will lead to a significant reduction in the incidence of grade 3 or 4 haematological adverse events.

### 2.2.1 Risks and benefits

Patients who stop CTX may be at a higher risk of experiencing HIV-related morbidity and malaria while those continuing on CTX may be at risk of drug side effects and / or drug to drug interactions.

# 3. SELECTION OF CENTRES/CLINICIANS

The study will be conducted in two existing MRC clinical research clinics in Entebbe Hospital and Masaka Hospital. Each of these clinics has recent experience in conducting a randomised trial of HIV treatment. Constitution of study teams will be finalised before commencement of study enrolment.

Training of study clinic teams on the new research protocol will be conducted one month before expected start of recruitment.

The co-Investigator responsible for conducting the study is a clinician with experience in ART related research. He will oversee the coordination of the clinical trial at both study sites, supervising the clinical care of patients, and ensuring compliance with the study protocol and GCP.

# 4. SELECTION OF PATIENTS

## 4.1 Patient inclusion criteria

a) HIV-infected patient taking cotrimoxazole for at least 6 months
b) Age 18 – 59 years
c) Documented intake of ART for at least 6 months
d) Clinically asymptomatic
e) 2 CD4 counts (not more than 6 months apart) ≥250 cells/mm$^3$ the most recent no more than 4 weeks prior to enrolment
f) Able to attend 3-monthly study clinics for appointments and in event of intercurrent illness.

## 4.2 Patient exclusion criteria

a) Acute illness (opportunistic infection or other co- morbidity).
*Patients will be considered for inclusion into the trial after resolution of the illness*
b) First trimester pregnancy. Pregnant women who *reach their second trimester of pregnancy could then be re-evaluated for inclusion into the trial.*
c) Known hypersensitivity to Cotrimoxazole

**Number and source of subjects**

It is expected that approximately 1000 patients will be recruited from each of the study sites.

Patients on long term ART care will be referred to the study sites from: MRC clinical research clinic in Entebbe; Entebbe Hospital ART clinic; TASO Entebbe centre ART clinic; Kitovu Hospital mobile ART clinic; Uganda Cares ART clinic – Masaka; TASO Masaka centre ART clinic.

## 4.3 Screening procedures and pre-randomisation investigations

Patients in participating centres who have been in ART care and on concurrent CTX prophylaxis for at least 6 months, will be given an information sheet about the COSTOP study and asked to give consent for screening.

Screening procedures: At screening, the following information will be recorded: Patients residence and contact details; Full medical history (including ART history, past WHO clinical stage 2, 3 and 4 events and malaria); Socioeconomic and behavioural history (including a sexual behaviour questionnaire, contraception, malaria prevention measures); full clinical examination (including weight and height).

Laboratory investigations to be carried out at screening will be: full blood count, malaria slide, CD4 count and pregnancy test (women of reproductive age); as well as any additional clinical tests as required by attending clinicians for the detection and management of HIV related conditions.

Enrolment procedures: Enrolment will be within 2 to 4 weeks of screening. Patient's eligibility for enrolment will be confirmed and repeat CD4 testing documented. A first indicative CD4 count will be not > 6 months prior to enrolment and the 2nd confirmatory enrolment CD4 count must have been done in an MRC lab not > 4 weeks prior to enrolment. Patients on ART and CTX prophylaxis, who are clinically well and have attained CD4 recovery to 250 cells/$mm^3$ and above will be eligible for enrolment.

Patients who are acutely ill at the time of screening may be rescreened for enrolment after resolution of the acute illness. Eligible women who are found to be in first trimester pregnancy at screening may be rescreened to ascertain eligibility to be enrolled in the trial after the end of the first trimester of pregnancy.

At the enrolment visit, clinical history and physical examination details will be recorded as well as results of all screening investigations within the preceding 4 weeks. A blood sample will be taken from which plasma will be stored for subsequent testing.

Fully informed signed consent will be obtained just before randomisation. Patients will be required to consent to be randomised to either continued ART plus CTX treatment or the placebo control group and indicate willingness to use an insecticide treated mosquito net. Separate consent will be sought for storage of blood specimens for subsequent testing related to HIV infection.

A trial register will be kept at clinical study sites with a record of all patients who are eligible and invited to join the trial. Those accepting will have name, date of birth (DOB), date of randomisation and trial number recorded. Those who refuse will have name, DOB, and reason for refusal recorded. The register will be kept in a secure place in each clinical site and will be the responsibility of the trial investigator at that site.

# 5. RANDOMISATION & ENROLMENT PROCEDURE

## 5.1 Randomisation practicalities

A randomisation schedule will be created by an independent statistician (based in the MRC Uganda Unit) using randomised blocks of variable size (see section 9.1). Two schedules, according to baseline CD4 count will be produced for each of the sites, Entebbe and Masaka.

Before enrolment screening forms and enrolment forms must have been completed. When a patient is confirmed to be eligible and has consented to being enrolled their details will be entered on the next available line of the enrolment register appropriate to their CD4 count to ascertain the trial number assigned to that participant which will be used on all trial documents and will identify the pre-labelled study medication (cotrimoxazole or placebo). This number is unique and should not be used for any other participant.

### 5.2 Randomisation codes and unblinding

The randomisation codes will be maintained by the independent trial statistician responsible for the schedule and a copy will be held by the trial pharmacist. Unblinding will be discouraged during treatment; if however, a trial clinician considers it necessary for a participant's allocated treatment to be unblinded this should first be discussed with the chief investigator or a clinical deputy. If unblinding is considered appropriate the reason for unblinding should be recorded in the unblinding register held in the trial office. This register should not include the details of the allocated treatment arm.

If it is considered appropriate to unblind a participant an unblinding form should be completed and sent to the independent statistician, or in their absence to the pharmacist. Unblinding information will be kept confidential to clinical staff responsible for the patient. The unblinding form will be entered on the data base.

### 5.3 Co-enrolment guidelines

Patients already enrolled in other studies, except for the Masaka discordant couples cohort, are not eligible for the COSTOP trial.

## 6. TREATMENT OF PATIENTS

### 6.1 Trial treatment

Eligible participants will be randomised to either continue prophylaxis with daily 960mg cotrimoxazole or to discontinue it while both groups continue to receive anti-retroviral treatment. Patients allocated to discontinue will receive daily matching cotrimoxazole placebo tablets. The follow-up schedule is given in detail in section 8.1.

### 6.2 Trial product(s)

Active cotrimoxazole (960 mg tablets) and matching placebo tablets will be from supplies obtained from CIPLA limited. These will packed and labelled by independent staff of the MRC Uganda AIDS Research Unit including the independent statistician responsible for the randomisation schedule. The label will include information on who the participant should contact in the event of the occurrence of severe adverse events which might be related to taking the study medication.

### 6.3 Dispensing

Patients will be seen at monthly intervals for the first three months and three-monthly thereafter. They will be provided with packs of 960 mg tablets (or placebo) sufficient for a 3 month period, with a few extra tablets to allow for the possibility of patients attending a few days late for their appointments. Patients will be requested to return their tablet packs with any unused tablets at scheduled clinic visits.

## 6.4 Modification of trial treatment

In the event of adverse drug reactions considered to be possibly related to study drug, study drug will be discontinued but **blinded allocation of study drug will be maintained, unless unblinding is required in the interest of patient safety** (for example in the case of a potential CTX related hypersensitivity reaction). The specific event will then be managed according to a pre established SOP. Adverse events will be graded according to the criteria set out in the appendix 3

In the event of a *confirmed CD4 count drop to below 250 cells/mm$^3$, placebo or active CTX will be discontinued and the subject **switched to open label CTX**. Subjects switched to open label CTX will continue to be followed until the end of the study.

If a *confirmed CD4 count drop to below 250 cells/mm$^3$ occurs following an adverse event determined to be hypersensitivity to Cotrimoxazole, this will prompt **unblinding of study drug allocation** to allow subsequent management of the patient.

*confirmed CD4 count drop = confirmed by a repeat test

## 6.5 Measures of adherence

Adherence to ART and CTX will be assessed using a standard adherence questionnaire (as used e.g. in the recently completed DART trial) and by returned pill count

## 6.6 ART and non-trial treatment

All participants will be treated with concomitant anti-retroviral treatment in line with the national programme guidelines. Other medication(s) or treatments as necessary for the management HIV related illnesses or other chronic disease conditions will be documented and evaluated for compatibility with administered study drugs.

# 7. ASSESSMENTS AND FOLLOW-UP

## 7.1 Schedule for follow-up / Flow Chart

*Patients will attend for scheduled post enrolment assessments at the study clinics monthly* during the first 3 months and 3-monthly thereafter.

Participants will also be encouraged to report to the study clinics whenever they are ill in between scheduled follow-up visits. Any missed visits within 2 weeks of a scheduled appointment will prompt tracing home visits by field workers and/or counselors to ascertain reasons for default. Similarly, failure to return to clinic within 14 days of a febrile episode or serious illness will lead to tracing of the study participant at home.

At each follow up assessment, the following will be performed:

- an adherence assessment,
- medical history and symptom check to detect intercurrent illness
- clinical examination and record of events since last visit
- full blood count, CD4 count, malaria slide, plasma storage as indicated by protocol schedule
- any or other clinical investigations as indicated by patient's clinical state

| Procedure | Assessment Time | | | | | | | |
|---|---|---|---|---|---|---|---|---|
| | Screening Visit ( week -2 to -4) | Enrolment Visit (week 0) | Week 4 | Week 8 | Week 12 | Week 24 | Every 3 months after | Every 6 months after |
| Consent for Screening & Blood Specimen Storage | x | | | | | | | |
| Consent for Enrolment | | x | | | | | | |
| History & Physical Examination[1] | x | x | x | x | x | x | x | |
| CD4 count | x | | | | x | x | | x |
| Full Blood Count | x | | | | x | x | x | |
| Pregnancy Test[3] | x | | | | | | | |
| Malaria slide | | x | x | x | x | x | x | |
| Plasma Storage | | x | | | | x | | x |
| Adherence assessment | | | x | x | x | x | x | |
| Study drug prescription/refill | | x | x | x | x | x | x | |

1. Doctor assessment. Including record of all clinical events and any adverse events since previous visit
2. CD4 counts at baseline, after first three months and then 6 monthly thereafter
3. Pregnancy test in all women of reproductive age at screening. Thereafter, only in event of amenorrhea.

## 7.2 Procedures for assessing efficacy

Assessment for CTX preventable events will be from clinical history and medical examination records performed by protocol trained research clinical staff, supplemented by laboratory measurements and ancillary clinical investigations. Recording of CTX preventable events will be made using the tables laid out by WHO on presumptive and definitive criteria for recognizing HIV-related clinical events in adults (15 years or older).[16] Clinical observations and attribution of end points will be verified by an independent endpoint review committee.

All laboratory tests will be carried out in the MRC laboratories, and results recorded on lab case record forms. Critical CD4 count measurements will be confirmed with a repeat test.

## 7.3 Procedures for assessing safety

Haematological (haematology, neutropenia and thrombocytopenia) events will be assesed through scheduled and clinically driven laboratory tests carried out in the MRC laboratories, and laboratory measurements recorded on lab case record forms. The DAIDS toxicity grading tables will be utilized to grade laboratory measured parameters.

Investigation of all patient-reported and clinically detected acute febrile episodes will include a blood slide for malaria parasites and the clinical outcome and severity of each malaria episode will be recorded separately onto a malaria episode form.

## 7.4 Loss to follow-up

All subjects will be followed up for a minimum of 18 months. In addition to protocol scheduled assessments, participants will also be encouraged to report to the study clinics whenever they are acutely ill. Any missed visits within 2 weeks of a scheduled appointment will prompt home visits by field workers and/or counselors to ascertain reasons for default. Similarly, failure to return to clinic within 14 days of an acute febrile episode or serious illness will lead to tracing of the participant at home.

## 7.5 Trial closure

On study follow up will be for a minimum of 18 months. The trial will be considered closed 36 months after the beginning of recruitment, after the study has been unblinded. Trial exit procedures will include documenting exit date and data on an exit form; and availing each patient with a clinical summary and a referral back to their respective national programme ART provision centres. Further clinical care of the enrolled patients will continue in the respective national ART provision clinics.

# 8. WITHDRAWAL OF PATIENTS

In consenting to the trial, patients are consenting to trial treatment, trial follow-up and data collection.

## 8.1 Withdrawal from trial intervention

Withdrawal from blinded trial allocation has been covered in section 6.4 and will be recorded on an unblinding form. Patients may in addition be withdrawn from study treatment for the following reasons:

i. Patient withdraws consent.
ii. Unacceptable toxicity.
iii. Intercurrent illness which prevents further treatment.
iv. Any change in the patient's condition which justifies the discontinuation of treatment in the clinician's opinion.

No additional patients will be recruited to replace those patients who withdraw from the study. Follow up of any patients so withdrawn from the study intervention and clinical data recording will continue unless the patient has explicitly also withdrawn consent for follow-up

## 8.2 Patient transfers

For patients moving from the study area, every effort will be made to ensure that their HIV clinical care is maintained at a convenient clinical centre and to the extent possible, to collect clinical status data which can be transcribed onto study CRFs.

## 8.3 Withdrawal from the trial completely

If a patient wishes to withdraw from trial treatment, study teams will nevertherless explain the importance of remaining on trial follow-up, or failing this of allowing routine follow-up data to be used for trial purposes. If the patient explicitly states their wish not to contribute further data to the study, the study team will document this on a withdrawal of consent form.

# 9. STATISTICAL CONSIDERATIONS

## 9.1 Method of Randomisation

Patients will be randomly allocated to one of the two treatment arms using random permuted blocks of variable size, with separate randomizations carried out in four strata, with the strata being defined by the four combinations of study site (Entebbe or Masaka) and baseline CD4 count (250-499 cells/mm3 vs. 500 cells/mm3 or above).

## 9.2 Outcome Measures

### 9.2.1 Primary

The co-primary outcome measures are

*Efficacy:* Time to the occurrence of the first clinical event (pre-defined CTX-preventable opportunistic infection or death).

*Safety:* Time to the occurrence of the first grade 3 or grade 4 haematological adverse event.

### 9.2.2 Secondary

The secondary outcome measures are

1. Incidence of all CTX preventable events
2. All cause mortality
3. Incidence of all clinical events and related events requiring hospitalisation
4. Incidence of all confirmed malaria episodes* asymptomatic and symptomatic
5. Severity and outcome of all confirmed malaria episodes* asymptomatic and symptomatic.
6. Incidence of grade 3 or grade 4 adverse events.
7. Mean change in CD4 count after 12 months on the trial.
8. Mean change in haematologic indices after 12 months on the trial.
9. Serious Adverse Events (SAEs)-according to ICH/GCP definitions.
10. Adherence to use of ART, trial drug and insecticide-treated mosquito nets.

* confirmed by positive parasitaemia on a blood slide.

## 9.3 Sample Size

The study aims to recruit an overall total of 2,000 patients in the two sites. The sample size calculations are based on the following assumptions:

1. The rate of clinical events (predefined opportunistic infections or death) in the control arm (subjects who continue with CTX prophylaxis) will be 10 per 100 PYO. This is based on an analysis of event rates among 1670 patients from the DART trial who had a confirmed CD4 count above 250 cells/mm$^3$. There were a total of 145 events in 2314 person years of observation giving an overall rate of 6.3 events per 100 person years of observation (PYO). The rate in the first year was much higher at 10.1 per 100 PYO (108 events in 1072 PYO). In general event rates in the DART trial were lower than rates reported from other studies in resource limited settings (including Uganda e.g. the recently completed cluster randomized trial in Jinja comparing home based and facility based care). This is believed to be at least partly due to the high levels of clinical care offered in the DART study. Thus in our sample size calculations we considered rates higher than those observed in DART.
2. Recruitment will be completed within 24 months and follow-up will continue for a further 18 months.
3. Loss to follow-up rate will be 4% per year.
4. Type I error probability (alpha) 0.05 (one-sided for non-inferiority).
5. A clinical event rate of 12.5% in the placebo arm would be considered non-inferior to that in the CTX arm (10%).
6. Power of 80% to detect non-inferiority of placebo to CTX i.e. the upper limit of the one-sided 95% confidence interval of the hazard ratio (HR) for placebo relative to CTX will be no greater than 1.25 (a relative increase of 25%, equivalent to an annual event rate in the placebo arm of 12.5% compared to 10% in the CTX arm) with probability 0.80 if placebo and CTX were truly equivalent.

Under these assumptions a total of about 2,000 patients would be required for the placebo *vs* CTX comparison. The target number of clinical events (CTX preventable event / death) is 494.

For the co-primary endpoint of the time to the first occurrence of a grade 3 or 4 haematological adverse event, a sample size of 1,000 subjects per arm will have approximately 85% power to detect as statistically significant at the 5% level a true hazard ratio of 2 for the arm that continues CTX prophylaxis relative to the arm that ceases CTX prophylaxis, if overall 10% of those in the arm that continues CTX prophylaxis experience such an event, and this sample size will have 90% power to detect as statistically significant at the 5% level a true hazard ratio of 2 if overall 12% of those in the arm that continues CTX prophylaxis experience such an event

## 9.4 Interim Monitoring and Analyses

The trial will be monitored by an Independent Data Safety and Monitoring Committee (IDMC). The IDMC will first meet 6 months after the first patient has been randomized; at this meeting they will decide on the frequency of future meetings, probably every 6-12 months. The IDMC will review the recruitment rate and event rate and assess whether the study is on schedule to meet its objectives. They will also review a summary of the malaria cases as adjudicated by the end point review committee, including the proportion of cases judged to be severe and the proportion of cases that were resolved without complications.

The IDMC will recommend stopping the trial if in their view the data provide statistical evidence beyond reasonable doubt of a difference between the two arms on the efficacy

endpoint of time to first CTX preventable event or death, as determined by the Haybittle-Peto approach to interim analysis; there would be such evidence of inferiority if the 99.9% confidence interval for the hazard ratio, for the experimental arm relative to the control arm, lies wholly above 1, while there would be such evidence of superiority if the 99.9% confidence interval for the hazard ratio lies wholly below 1.

The COSTOP TSC will then decide whether to amend or stop the trial before the end of the planned follow-up. The decision will take into account the severity of the observed events.

The IDMC will also report to the COSTOP TSC if the upper limit of the 95% confidence intervals for the event rate for the composite endpoint of cotrimoxazole preventable event or death, in both arms is less than 1 per 100 person years of observation.

## 9.5 Analysis Plan (brief)

The primary analysis will compare the experimental (placebo) group to the control (continued cotrimoxazole prophylaxis) group in terms of

(a) Time to first CTX-preventable event or death
(b) Time to first haematological grade 3 or 4 adverse event.

For the time to first CTX-preventable event or death the analysis will test for non-inferiority, hence the main analysis will be a per protocol analysis ( in non-inferiority studies an intention-to-treat analysis is not conservative). Time to event methods will be used (Kaplan Meier plots, stratified log rank test and Cox proportional hazards regression). Non-inferiority will be tested by estimating the hazard ratio for the experimental arm versus the control arm and finding the two-sided 90% confidence interval. The experimental arm will be deemed to be non-inferior to the control arm if the upper limit of the confidence interval is less than 1.25. As a form of sensitivity analysis, an intention to treat analysis will be carried out including all subjects who took at least one dose of the blinded study medication.

For the time to first grade 3 or 4 haematological adverse event, an intention to treat analysis will be carried out, also using time to event analysis (Kaplan Meier plots, stratified log rank test and Cox proportional hazards regression), with superiority tested using the stratified log rank test.

Similar methods will be used for the secondary outcome measures; since these are not analysed as non-inferiority endpoints, an intention-to-treat analysis will be used in all cases. A full analysis plan will be developed under version control before the final data lock, and agreed by the TSC and the DSMC.

# 10. TRIAL MONITORING

COSTOP is a Phase IV trial which does not involve the use of a new investigational drug. This informs the decision to have low-level monitoring of the trial.

## 10.1 Monitoring in the Data Section at MRC/UVRI Entebbe

The study database will be set up using an SQL platform with an MS-ACCESS front end. All data will be double-entered and data validated before being uploaded into the database. The data entry screens will incorporate a number of range checks and logical skips as appropriate. Data stored in the database will be checked for missing or unusual values (range checks) and checked for consistency within participants over time. If any such problems are identified, a photocopy of the problematic CRF(s) will be returned to the site concerned for checking and confirmation or correction, as appropriate. The amended version should be returned to the data section and the site's copy should also be amended. The trial data manager will send reminders for any overdue and missing data.

The Masaka site will be responsible for maintaining its own database and for timely (twice monthly) transfer of checked data to Entebbe for merging with the main trial database.

## 10.2 Clinical Site Monitoring

### 10.2.1 Direct Access to Data

The COSTOP TMG will allow ethics committee review by the UVRI Science and Ethics Committee and the UNCST if required and will also allow the NDA to carry out regulatory inspections if required, by providing access to source data/documents. The informed consent process will include patients' consent for such monitoring reviews of the data.

### 10.2.2 Confidentiality

Each site will keep a separate patient record database which links the patient's name and contact details to his or her study number, for purposes of patient follow-up in the case of missed visits. However the study database will include only the patient study number. Individual patients will not be identified in publications and presentations from the trial.

### 10.2.3 Quality Assurance and Quality Control of Data

Data monitoring will be carried out using cross site monitoring i.e. one or more trial staff members from Masaka will monitor the data from the Entebbe site and vice versa, under the oversight of the TMG. The cross-site monitors may consult and/or copy source records (clinical notes, laboratory values) in order to do this. Such information will be treated as strictly confidential and will in no circumstances be made publicly available. The monitoring will adhere to Good Clinical Practice Guidelines (based on ICH guidelines). The following data should be verifiable from source documents: all signed consent forms; dates of visits including laboratory results; eligibility and baseline values for all study subjects; all clinical endpoints; all serious / severe adverse events; an ongoing 5% random sample of routine patient clinical and laboratory data; dates drug dispensed; drug adherence (both for ART and for blinded study medication) and details of all concomitant medication. In the event that the random sample monitoring identifies concerns about data quality additional checks will be put in place until such time as any problems identified have been resolved.

# 11. SAFETY REPORTING

ICH GCP requires that both investigators and sponsors follow specific procedures when notifying and reporting adverse events/reactions in clinical trials. These procedures are described in this section of the protocol.

## 11.1 Definitions

The definitions of the EU Directive 2001/20/EC Article 2 based on ICH GCP apply in this trial protocol. These definitions are given in the Table 1.

**Table 1: Definitions**

| Term | Definition |
|---|---|
| **Adverse Event (AE)** | Any untoward medical occurrence in a patient or clinical trial subject to whom a medicinal product has been administered including occurrences which are not necessarily caused by or related to that product. |
| **Adverse Reaction (AR)** | Any untoward and unintended response to an investigational medicinal product related to any dose administered. |
| **Unexpected Adverse Reaction (UAR)** | An adverse reaction, the nature or severity of which is not consistent with the information about the medicinal product in question set out in the summary of product characteristics (or Investigator brochure) for that product. |
| **Serious Adverse Event (SAE) or Serious Adverse Reaction (SAR) or Suspected Unexpected Serious Adverse Reaction (SUSAR)** | Respectively any adverse event, adverse reaction or unexpected adverse reaction that:<br>• results in death<br>• is life-threatening*<br>• requires hospitalisation or prolongation of existing hospitalisation**<br>• results in persistent or significant disability or incapacity<br>• consists of a congenital anomaly or birth defect |

### 11.1.1 Clarifications and Exceptions

*The term 'life-threatening' in the definition of 'serious' refers to an event in which the patient was at risk of death at the time of the event; it does not refer to an event which hypothetically might have caused death if it were more severe.

**Hospitalisation is defined as an inpatient admission, regardless of length of stay, even if the hospitalisation is a precautionary measure for continued observation. Hospitalisations for a pre-existing condition (including elective procedures that have not worsened) do not constitute an SAE.

Medical judgement should be exercised in deciding whether an AE/AR is serious in other situations. Important AE/ARs that are not immediately life-threatening or do not result in death or hospitalisation but may jeopardise the subject or may require intervention to prevent one of the other outcomes listed in the definition above, should also be considered serious.

### 11.1.2 Trial Specific Exceptions to Expedited SAE Notification and Reporting:

Disease progression or death as a result of disease progression are not considered to be SAEs and should be reported on the Death Report Form.

Due to the seriousness of the disease in this study, the following situations that fulfill the definition of an SAE are excluded from expedited notification on an SAE form and should be reported on the specific case report form.

- Elective hospitalisation to simplify treatment or procedures
- Elective hospitalisation for pre-existing conditions that, in the investigator's opinion, have not been exacerbated by trial treatment.

Instances of the following treatment-related toxicities that result in hospitalisation for symptom control are also excluded from expedited reporting and should be recorded in the Toxicity section of the Treatment/Progress Forms. Life-threatening or fatal events should still be reported on the SAE form:

- Hypersensitivity reactions
- Haematologic toxicities (anaemia, neutropenia, thrombocytopenia)

## 11.2 Institution/Investigator Responsibilities

All non-serious AEs/ARs, whether expected or not, should be recorded in the toxicity (symptoms) section of the case report form and sent to MRC/UVRI Entebbe within one month of the form being due. SAEs/SARs should be notified to MRC/UVRI Entebbe.

The severity (i.e. intensity) of all AEs/ARs (serious and non-serious) in this trial should be should be graded using the Division of AIDS Table for Grading the severity of Adult and Paediatric Adverse Events Version 1.0, Decenber 2004; Clarification August 2009.
A version summarized for this trial is available in Appendix x.

A flowchart is given at the end of this section to help explain the notification procedures. Any questions concerning this process should be directed to the MRC CTU in the first instance.

### 11.2.1 Investigator Assessment

**(a) Seriousness**
When an AE/AR occurs the investigator responsible for the care of the patient must first assess whether the event is serious using the definition given in Table 1. If the event is serious and not exempt from expedited reporting, then an SAE form must be completed and the MRC/UVRI Entebbe notified.

**(b) Causality**
The Investigator must assess the causality of all serious events/reactions in relation to the trial therapy using the definitions in Table 2. There are 5 categories: unrelated, unlikely, possible, probable and definitely related. If the causality assessment is unrelated or unlikely to be related the event is classified as a SAE. If the causality is assessed as either possible, probable or definitely related then the event is classified as a SAR.

**Table 2: Definitions of causality**

| Relationship | Description | Event Type |
|---|---|---|
| **Unrelated** | There is no evidence of any causal relationship | SAE |
| **Unlikely** | There is little evidence to suggest there is a causal relationship (e.g. the event did not occur within a reasonable time after administration of the trial medication). There is another reasonable explanation for the event (e.g. the patient's clinical condition, other concomitant treatment). | SAE |
| **Possible** | There is some evidence to suggest a causal relationship (e.g. because the event occurs within a reasonable time after administration of the trial medication). However, the influence of other factors may have contributed to the event (e.g. the patient's clinical condition, other concomitant treatments). | SAR |
| **Probable** | There is evidence to suggest a causal relationship and the influence of other factors is unlikely. | SAR |
| **Definitely** | There is clear evidence to suggest a causal relationship and other possible contributing factors can be ruled out. | SAR |

## (c) Expected

If the event is a SAR the Investigator must assess the expectedness of the event. The definition of an unexpected adverse reaction (UAR) is given in Table 1. Potential toxicities associated with co-trimoxazole are:

- Skin rash / hypersensitivity reaction
- Bone marrow toxicity (haematologic toxicity – anaemia, neutropenia
- Hepatotoxicity

For a listing of expected toxicities associated with antiretroviral therapy and their management, please refer to appendix 5.

If a SAR is assessed as being unexpected it becomes a SUSAR.

## (d) Notification

MRC/UVRI Entebbe should be notified within one working day of the investigator becoming aware of an event that requires expedited reporting. Investigators should notify the MRC/UVRI of all SAEs occurring from the time of randomisation until 30 days after the last protocol treatment administration. SARs and SUSARs must be notified to the MRC/UVRI indefinitely (i.e. no matter when they occur after randomisation).

### Notification Procedure:

1. The SAE form must be completed by the Investigator (named on the signature list and delegation of responsibilities log who is responsible for the patient's care), with due care being paid to the grading, causality and expectedness of the event as outlined above. In the absence of the responsible investigator the form should be completed and signed by a member of the site trial team. The responsible investigator should subsequently check the SAE form, make changes as appropriate, sign and then re-fax to MRC/UVRI as soon as possible. The initial report shall be followed by detailed, written reports as appropriate.

2. Send the SAE form as an email attachement to MRC/UVRI Entebbe
   Email: ronnie.kasirye@mrcuganda

3. Follow-up: Patients must be followed-up until clinical recovery is complete and laboratory results have returned to normal or baseline, or until the event has stabilised. Follow-up should continue after completion of protocol treatment if necessary. Follow-up information should be noted on a further SAE form by ticking the box marked 'follow-up' and sending to MRC/UVRI Entebbe as information becomes available. Extra, annotated information and/or copies of test results may be provided separately. The patient must be identified by trial number, date of birth and initials only. The patient's name should not be used on any correspondence.

4. Staff at the institution must notify the ethics committee of the event (as per the institutions standard local procedure).

## 11.3 MRC / UVRI Responsibilities

Medically qualified staff at the MRC/UVRI and/or the Chief Investigator (or a medically qualified delegate) will review all SAE reports received. The causality assessment given by the site Investigator cannot be overruled and in the case of disagreement, both opinions will be provided in any subsequent reports.

MRC / UVRI is responsible for the reporting of SUSARs and other SARs to the regulatory authorities and the research ethics committees as appropriate.

MRC/UVRI will also keep all investigators informed of any safety issues that arise during the course of the trial.

**SAE NOTIFICATION**

Within one working day of becoming aware of an SAE, please send a completed SAE form to the Study Coordinator at MRC / UVRI on:

**Email: ronnie.kasirye@mrcuganda.org**

## Figure 3: Safety Reporting Flowchart

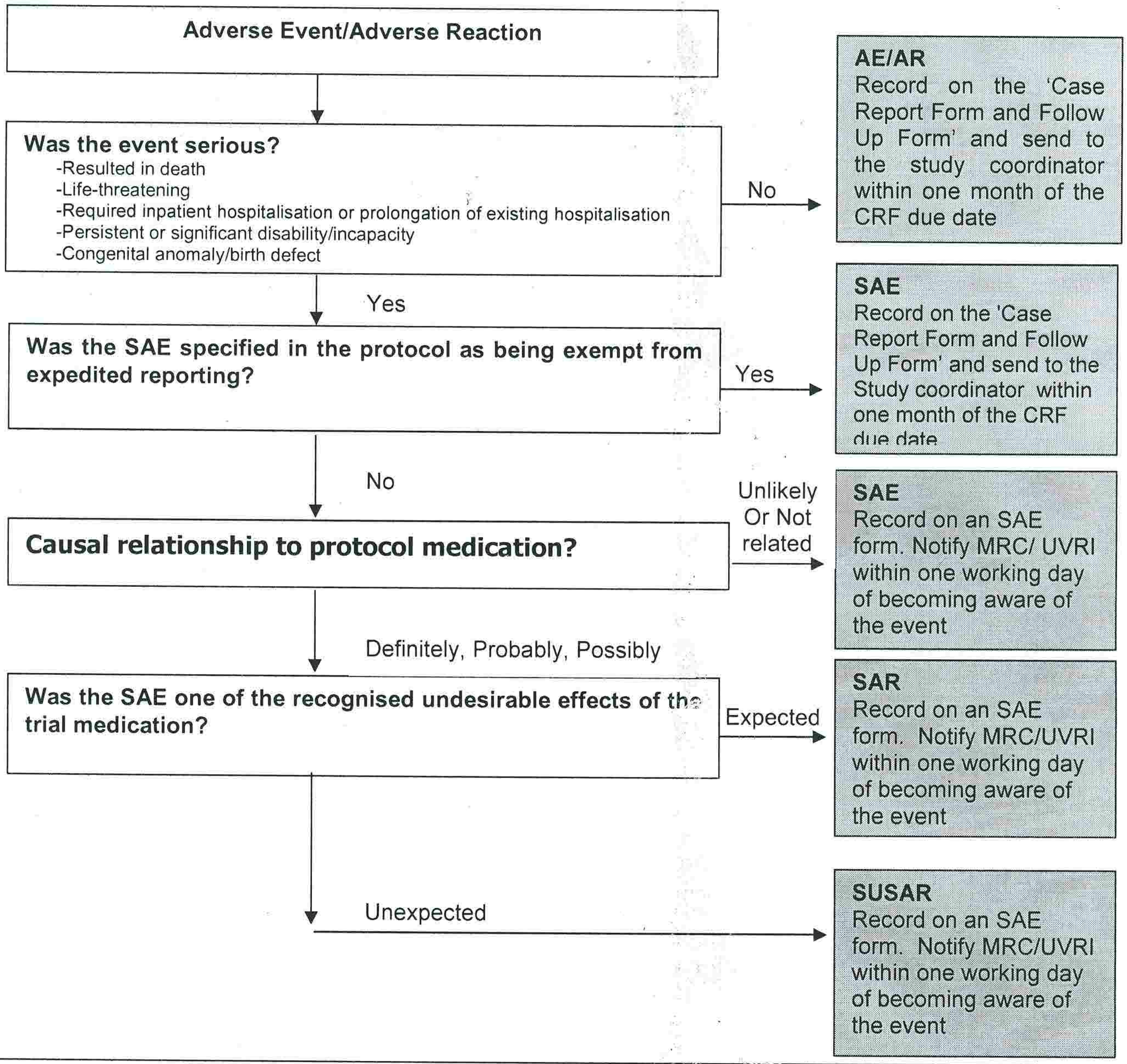


**CRF:** Case report form
**SAE:** Serious adverse event
**SAR:** Serious adverse reaction
**SUSAR:** Suspected unexpected serious adverse reaction

# 12. ETHICAL CONSIDERATIONS AND APPROVAL

## 12.1 Ethical considerations

The study will abide by the principles of the Declaration of Helsinki.

Patients who stop CTX may be at a higher risk of experiencing HIV-related morbidity and malaria while those continuing on CTX may be at risk of drug side effects and / or drug to drug interactions. The trial will be monitored by an Independent Data Monitoring Committee (IDMC) who will advise the researchers to modify or terminate the trial if required.

The trial we propose will be hospital clinic based allowing close clinical supervision and care of patients. Patient information will include education on recommended prevention of malaria and additional preventive measures against malaria such as provision and ensuring use of insecticide treated bednets for the study participants will be practiced.

A unique trial number will identify all laboratory specimens, case record forms, and other records and no names will be used, in order to maintain confidentiality. All records will be kept in locked locations. Clinical information will not be released without written permission, except as necessary for monitoring by the trial monitors.

Participants will be free to withdraw from the trial after enrolment if they do so wish, without compromising their care.

## 12.2 Ethical approval

This document, along with any subsequent modifications and with the sample informed consent documents will be reviewed by the UVRI Science and Ethics Commitee and Uganda national Council of Science and Technology.

Regulatory approval for conduct of the trial will be obtained from the National Drug Regulatory Authority.

Ethical approval for this amended protocol will be sought from the UVRI Science and Ethics Committee and from the Uganda National Council for Science and Technology. Additional Regulatory clearance will be sought from the National Drug Regulatory Authority.

# 13. INDEMNITY

MRC will give sympathetic consideration to claims for non-negligent harm suffered by a person as a result of trial or other work supported by MRC. This does not extend to liability for non-negligent harm arising from conventional treatment where this is one arm of a trial. MRC acts as its own insurer and does not provide cover for non-negligent harm in advance for participants in MRC-funded studies.

Where studies are carried out in a hospital, the hospital continues to have a duty of care to a patient being treated within the hospital, whether or not the patient is participating in an MRC-supported study. MRC does not accept liability for any breach in the hospital's duty of care, or any negligence on the part of employees of hospitals.

# 14. TRIAL COMMITTEES

The trial will be overseen by a Trial Steering Committee, Trial Management Group and Independent Data Monitoring Committee. An Endpoint Review Committee will also be appointed. The Trial Steering Committee and Independent Data Monitoring Committee will be given an opportunity to comment on a pre-final version of the trial protocol.

## 14.1 Trial Management Group (TMG)

The Trial Management Group (TMG) will be comprised of the Chief Investigator, other lead investigators (clinical and non-clinical) and the managers of the participating TASO centres. The TMG will be responsible for the day-to-day running and management of the trial and will meet monthly in person or by teleconference.

## 14.2 Trial Steering Committee (TSC)

The Trial Steering Committee (TSC) will consist of an independent chairman and at least two additional independent members. The role of the TSC is to provide overall supervision for the trial and provide advice through its independent chairman. The TSC receives recommendations from the IDMC and must decide on whether to act upon those recommendations; it has the authority to prematurely close the trial. A TSC Charter will be developed.

## 14.3 Independent Data Monitoring Committee (IDMC)

The Independent Data Monitoring Committee whose responsibilities will be to safeguard the interests of trial participants, assess the safety and efficacy of the trial intervention and monitor its overall conduct. The IDMC can recommend modification or premature closure of the trial to the TSC.

The IDMC will usually meet every 4-6 months, the frequency of meetings to be determined by the committee. IDMC meetings will consist of open and closed sessions, the terms of reference will be described within the IDMC charter. Details of the interim analysis and monitoring are provided in the IDMC charter and in section 9.4.

## 14.4 Endpoint Review Committee (ERC)

An Endpoint Review Committee, consisting of independent clinicians will adjudicate on clinical events, blind to allocated treatment.

# 15. PUBLICATION

The COSTOP Trial Management Group (TMG) will develop guidelines for the preparation of papers (including abstracts) for presentation at national and international meetings, as well as the preparation of manuscripts for peer-reviewed publication. Any publication or presentation during the active phase of the study must have the prior approval of the TSC. All publications will acknowledge the MRC as funder of the study. The members of the TSC and IDMC should be listed with their affiliations in the Acknowledgements of the main publication. The COSTOP TMG is the custodian of the data; the data are not the property of individual participating investigators.

# 16. PROTOCOL AMENDMENTS

The previous protocol for this study which was approved by the UVRI Science and Ethics committee was **Version 3.1 of 03.08.2007**, entitled 'Protocol for Cotrimoxazole prophylaxis Cessation Study among stabilized HIV-infected adult patients on Highly Active Anti-Retroviral Therapy (HAART) in Entebbe, Uganda.'

There have been some changes to the study design that have arisen as a result of comments from the MRC Review Boards in London who reviewed our application for funding. One recommendation was that we collaborate with the MRC Clinical Trial Unit (CTU) in London; hence Professor Andrew Nunn of CTU is a co-investigator on the study.

This revised protocol is has also been re-formatted using the MRC CTU protocol template and this has resulted in a re-organization of the material and a clearer protocol.

The following are the substantive changes to the protocol:

A. Change from double randomisation to single randomisation
B. Modification of study objectives
C. Revision of non-inferiority hazard ratio
D. Modification of inclusion exclusion criteria
E. Inclusion of stopping guidelines for the IDMC

Detail of amendments:

A. In the earlier protocol there were potentially two separate randomizations, as described in Version 3.1 on page 6:

***1st randomisation:*** *Participants who have been on HAART for at least 3 months and who have a confirmed CD4 count between 200 cells / mm3 and 350 cells / mm3 will be randomized to continue prophylaxis with active cotrimoxazole or to cease prophylaxis with active cotrimoxazole but continue with ingestion of placebo cotrimoxazole daily.*
***2nd randomization:*** *Participants who achieve a confirmed CD4 count of 350 cells / mm3 and above while on HAART will be randomized to continue prophylaxis with active cotrimoxazole or to cease prophylaxis with active cotrimoxazole but continue with ingestion of placebo cotrimoxazole daily. Some patients will have participated already in 1st randomization but others will be entering the trial at this stage for the first time.*

This has now been changed to a single randomization on the advice of several referees who felt that the new design would both be simpler to implement and also enable us to better answer the main research question, namely whether it is safe to stop CTX prophylaxis in patients who are stabilized on ART. The new design is given below for comparison.

Eligible patients will be HIV +ve adults aged 18 years and above who are stable on ART and have confirmed sustained CD4 restoration to250 cells/mm$^3$ and above with no contraindication to being randomised to placebo or control groups and who are able to attend designated study clinics. Patients who are eligible will be randomised 1:1 to the experimental or control group:

- Experimental group: one oral tablet daily of placebo CTX daily
- Standard/Control group: one oral tablet of 960 mg of CTX daily

All patients will continue to take their ART.
The trial schema has also been changed accordingly.

B. The objectives of the study have been modified accordingly.
The previous objectives were:

1. *To evaluate verified morbidity effects of cessation or continuation of cotrimoxazole prophylaxis among HAART patients following successful immune restoration (CD4-guided).*
2. *To compare haematological changes associated with cessation or continuation of cotrimoxazole prophylaxis in the study context.*
3. *To determine time from CD4 of 200 to 350 /mm3 among patients stopping or continuing with cotrimoxazole prophylaxis in HIV-infected adults who have stabilized on HAART.*

The new objectives are:

Principle Question: Can long-term primary and secondary prophylaxis with cotrimoxazole be safely discontinued among African adults on antiretroviral therapy who have achieved sustained immune restoration (measured as a confirmed increase in CD4 count to 250 or more cells/mm3)?

The objective of the study is to assess whether, in patients with CD4 count 250 or more cells/$mm^3$, the intervention regimen of discontinuation of CTX prophylaxis is (a) not inferior to the control regimen in which prophylaxis with CTX is continued and (b) superior with respect to the incidence of haematological adverse events.. i.e. the hypothesis is that stopping CTX will not lead to an excess of clinical events (predefined opportunistic infections or death) but will lead to a significant reduction in the incidence of grade 3 or 4 haematological adverse events.

C. The initial proposal had a non-inferiority hazard ratio of 1.20; we have increased this to 1.25. The new estimated overall event rate of 10 per 100 PYO is lower than the previous estimate of 20 per 100 PYO; this was based on an analysis of patients in the DART trial who had reached a confirmed CD4 count of 250 cells/mm3. The DART data were not available at the time that Protocol 3.1 was written. Thus although the protocol now allows a larger relative difference in the number of events in order to conclude non-inferiority, the absolute difference in the number of events that will lead to a conclusion of non-inferiority is smaller. Under the assumptions of Protocol 3.1 we could conclude non-inferiority with a maximum difference of 4.0 events per 100 PYO, whereas under the current assumptions we would conclude non-inferiority with a maximum difference of 2.5 events per 100 PYO. It is worth noting that in the CTX cessation trial in Moshi, Tanzania, the non-inferiority hazard ratio is 1.66.

D. The inclusion / exclusion criteria have been modified and include a higher baseline CD4 count of 250 cells/mm3, compared to the value of 200 cells/mm3 used in Protocol 3.1. This is related to the change in ART treatment guidelines currently used in Uganda, and is also slightly more conservative than the current guidelines for stopping CTX prophylaxis in developed countries. The inclusion and exclusion criteria in version 3.1 and the current protocol are given below.

*Protocol 3.1 Inclusion:*

- *Consenting HIV-infected participant aged between 16-59 years,*
- *Resident within 15-40 kms of study clinics*
- *Regularly attending TASO or UVRI study clinics*
- *Documented HAART intake for at least 3 months*
- *Clinically healthy and stable*
- *Confirmed CD4 count of 200 cells/$mm^3$ and above.*

*Protocol 3.1 Exclusion:*

- *Acutely ill patients with opportunistic or other infections*
- *Patients already enrolled in other HAART trials (e.g DART trial)*
- *First trimester pregnancy at enrolment**
- *Clinical and immunological evidence of HAART treatment failure*
- *Unable to attend study clinics regularly*
- *Hypersensitivity to cotrimoxazole*

**Eligible pregnant patients beyond the first trimester will be enrolled into the trial and they will be maintained on the recommended Intermittent Presumptive Therapy for malaria. They will be referred for appropriate obstetric care to Entebbe hospital.*

Current Protocol Patient inclusion criteria:

- HIV-infected patient taking cotrimoxazole for at least 6 months
- Age 18 – 59 years
- Documented intake of ART for at least 6 months
- Clinically asymptomatic
- 2 CD4 counts (not more than 6 months apart) ≥250 cells/mm3 the most recent no more than 4 weeks prior to enrolment
- Able to attend 3-monthly study clinics for appointments and in the event of intercurrent illness occurring.

Current Protocol Patient exclusion criteria

- Acute illness (opportunistic infection or other co- morbidity).
- Patients will be considered for inclusion into the trial after resolution of the illness
- First trimester pregnancy. Pregnant women who reach their second trimester of pregnancy could then be re-evaluated for inclusion into the trial.
- Known hypersensitivity to cotrimoxazole

E. At the suggestion of the MRC Review Board we have specified stopping guidelines for the IDMC in section 9.4. These were not included in Protocol 3.1 .

# 17. REFERENCES

1. Uganda Ministry of Health. National Policy Guidelines for Cotrimoxazole Prophylaxis for people with HIV/AIDS. 2005.

2. Wiktor S, Sassan-Morokro M, Grant A et al. Efficacy of trimethoprim-sulphamethoxzaole prophylaxis to decrease morbidity and mortality in HIV-1-infected patients with tuberculosis in Abidjan, Cote d'Ivoire: a randomised trial. The Lancet 1999;353:1469-1475

3. Anglaret X, et al. Early chemoprophylaxis with trimethoprim-sulphamethoxazole for HIV-1-infected adults in Abidjan, Cote d'Ivoire: a randomised trial. The Lancet 1999;353:1463-1468

4. Maynart M, et al. Primary prevention with cotrimoxazole for HIV-1-infected adults: results of the pilot study in Dakar, Senegal. Journ Acquir Immune Def Syndr 2001;130-136

5. Badri M, Ehlirch R, Wood R, Maartens G. Initiating co-trimoxazole prophylaxis in HIV-infected patients in Africa: an evaluation of the provisional WHO/UNAIDS recommendations. AIDS 2001;15:1143-1148

6. Mermin J, Lule J, Ekwaru J et al. Effect of co-trimoxazole prophylaxis on morbidity, mortality, CD4-cell count, and viral load in HIV infection in rural Uganda. The Lancet 2004;364:1428-1434

7. Mwangulu F, Floyd S, Crampin A et al. Cotrimoxazole prophylaxis reduces mortality in human immunodeficiency virus-positive tuberculosis patients in Karonga District, Malawi. Bull World Health Org 2004;82:354-363

8. Nunn A, Mwaba P, Chintu C, Mwinga A, Darbyshire J, Zumla A. Role of co-trimoxazole prophylaxis in reducing mortality in HIV infected adults receiving treatment for tuberculosis: randomized clinical trial. Brit Med Journ 2008; 337:a257

9. World Health Organization. Guidelines on cotrimoxazole prophylaxis for HIV-related infections among children, adolescents and adults in resource limited settings. Recommendations for a public health approach. 2006

10. Moh R, Danel C, Sorho S et al. Haematological changes in adults receiving a Zidovudinecontaining HAART regimen in combination with cotrimoxazole in Cote d'Ivoire.
Antivir 2005; 10(5): 615-24.

11. Uganda Ministry of Health. National Antiretroviral Treatment and Care Guidelines for Adults and Children. 2nd Edition July 2008.

12. DHHS Guidelines for the Prevention of Opportunistic Infections Among HIV-infected Persons- 2002. http://aidsinfo.nih.gov/contentfiles/OIpreventionGL.pdf

13. Lowrance D et al. Lower early mortality rates among patients receiving antiretroviral treatment at clinics offering cotrimoxazole prophylaxis in Malawi. JAIDS 2007;46:56-61

14. Campbell J, Tappero J et al. HIV-infected Ugandans on HAART with CD4 cell counts over 200 cells/mm3 who discontinue cotrimoxazole have increased risk of malaria and diarrhoea. 2008 HIV Implementers' Meeting, Kampala, Uganda. www.hivimplementers.com/

15. Malaria Control Programme, Ministry of Health. Uganda Malaria Control Strategic Plan 2005/06-2009/10. http://www.rollbackmalaria.org/countryaction/nsp/uganda.pdf

# 18. APPENDICES

APPENDIX 1: PATIENT INFORMATION SHEET ........ 35
APPENDIX 2: CONSENT FORMS........ 38
APPENDIX 3: TOXICITY TABLE........ 40
APPENDIX 4: CLINICAL EVENT CRITERIA ........ 52
APPENDIX 5: TOXICITY MANAGENT TABLES ........59
APPENDIX N: CASE REPORT FORMS........ 58

# APPENDIX 1: PATIENT INFORMATION SHEET

**Study Title: Safety of discontinuing Cotrimoxazole Prophylaxis among African adults on ART. A randomised controlled trial. COSTOP**

You are being invited to take part in a research study. Before you decide it is important for you to understand why the research is being done and what it will involve. Please take time to read the following information carefully and discuss it with others if you wish. Ask us if there is anything that is not clear or if you would like more information. Take time to decide whether or not you wish to take part. Thank you for reading this.

### What is the purpose of the study?

In Uganda (and other countires in Africa), it is advised for all HIV positive persons to take the medicine Cotrimoxazole (commonly known as Septrin), every day. This advice is based on information from research studies that were carried out in Uganda and other countries in Africa, which showed that taking Septrin daily leads to a reduction in the frequency of common illnesses that affect persons with HIV and leads to an overall improvement in their health. Septrin is not an anti-HIV medicine, but when taken daily, is a medicine that "prevents" other infections from affecting people with HIV. The studies on Septrin "preventive treatment" were all done in the period before the specific anti-HIV medicines (known as antiretroviral medicines) became widely available in Africa and the studies were carried out in patients who were not yet taking antiretroviral medicines.

Since antiretroviral medicines themselves lead to restoration of the body's immune function so that treated patients with HIV infection become less susceptible to the common HIV-related illnesses which Septrin prevents, it is probably not necessary to continue to take Septrin daily while one is also talking antiretroviral medicines. Doctors are furthermore concerned that continuing to take Septrin daily for an indefinite time, at the same time as taking antiretroviral medicines for life, presents an additional burden of tablets on patients, may increase the risk of some side effects and increases the overall costs of treatment.

It is however, not known for sure, if it is safe to discontinue taking Septrin and when it would be safe to discontinue taking Septrin among African patients who are receiving antiretroviral medicines and who have responded well to their antiretroviral treatment.

The COSTOP study aims to find out whether and when it is safe to discontinue Septrin as a "preventive treatment" once patients have started to receive antiretroviral medicines and have responded well to antiretroviral medicines.

### Why have I been chosen?

You have been approached today to ask you to join this study because you have been on antiretroviral medicines for 6 months or longer, a duration of treatment that indicates you are stable on your antiretroviral medicines and because your health state in the recent past has not been of concern to your doctors. 2000 patients altogether will be enrolled in this study.

### How can I join the study?

After reading this information sheet you will be asked to give your written agreement to be screened, which will involve being seen by the doctor and giving a blood sample. After two weeks you will be told whether you are eligible for the study and asked to sign a study consent form if you choose to participate.

**Do I have to take part?**

It is up to you to decide whether or not to take part. If you do decide to take part you will be given this information sheet to keep and be asked to sign a consent form. If you decide to take part you are still free to withdraw at any time and without giving a reason. A decision to withdraw at any time, or a decision not to take part, will not affect the standard of care you receive.

**What will happen to me if I take part?**

The research will last 3 years. After you have given your written agreement to participate in the study, you will be requested to attend the study clinic every month for the first three months and every 3 months thereafter, as well as whenever you are ill. A transport refund will be provided to you to attend the study clinic appointments and an insecticide treated mosquito bednet will be provided to you.

At clinic visits, study nurses, field workers, counsellors and doctors will separately ask you questions about your health and your household. You will be requested to give a blood sample (5-10 mls / equivalent to 1 tablespoonful) for study tests.

Once you have agreed to enter the trial, you will **continue taking your usual antiretroviral medicines** but will be required to stop taking the Septrin tablets that you have been taking. Instead, you will be allocated to one of two treatment groups:

- Cotrimoxazole treatment group ( will take one cotrimoxazole tablet daily)
- Cotrimoxazole placebo group (will take one cotrimoxazole placebo tablet daily)

There are equal chances of either treatment group being the one you will receive. Allocating treatment this way is done by the computer and means that the groups of people getting each treatment will be similar.

Neither you nor your doctor will know which treatment group you are in because all the treatments will look the same (although, if your doctor needs to find out he/she can do so). The placebo treatment is a tablet which looks like the cotrimoxazole treatment but it is not. It contains no active ingredient. "Cotrimoxazole" is the same medicine as "Septrin".

Study field workers will visit your home (if you have no objection) in the event that you do not return for clinic review visits and occasionally to check on mosquito bednet use

**What are the possible disadvantages and risks of taking part?**

Patients who stop taking cotrimoxazole may be at a slightly higher risk of experiencing fevers and malaria. The study clinic team is accessible to you in event of any ill health and will request that you sleep under a mosquito net. You may experience some mild discomfort from drawing of study blood samples.

**What are the possible benefits of taking part?**

The information we get from this study will help us to treat future patients with HIV infection better. There is however no anticipated direct individual benefit to yourself.

**What happens when the research study stops?**

After the research stops you will continue to receive your antiretroviral medicines from your current provider and the study team will advise on you on further additional Septrin according to the findings of this study.

**Will my taking part in this study be kept confidential?**

If you consent to take part in the research, your medical information may be seen by staff of the MRC Entebbe for purposes of analysing the results. It may also be looked at by people from regulatory authorities to check that the study is being carried out correctly. Your name, however, will not be displayed on any of this information outside the study clinic and you cannot be recognised from it.

This study is being carried out by the MRC/UVRI Research Unit on AIDS and will take place at MRC in Entebbe and MRC in Masaka. The study is funded by MRC (UK).

Please feel free to ask the doctors, nurses and counsellors about anything that you feel you have not understood. You can also contact the study teams on the telephone numbers below.

**MRC Entebbe: ....................................**
**MRC Masaka: ............................................**

This study was approved by the UVRI science and ethics committee, so for any questions about your rights as a study participant please contact

**The chairman**
**UVRI Science and Ethic Committee**
**Uganda Virus Research Institute**
**P.O Box, Entebbe**
**Telephone: 0414320385/6**

Thank you for taking the time to read this form and for considering joining the study.

# APPENDIX 2: CONSENT FORMS

## 2.1 CONSENT FOR SCREENING

Centre: Entebbe ❍Masaka ❍

| | | | |
|---|---|---|---|
| Date of screening | __ __ / __ __ / 2 0 __ __ | Clinic / hospital number | __ __ __ __ __ __ __ __ __ __ |
| Date of birth | __ __ / __ __ / 1 9 __ __ | Sex: | Male ☐ Female ☐ |

I have read the information sheet for the COSTOP study and agree to be screened to assess my eligibility for enrolment into this trial.

I understand this will involve being seen by the doctor, who will ask questions about my current health, my past health and my household, as well as having some blood taken.

| Individuals signature / thumb print | Name | Date |
|---|---|---|
| | | |
| WITNESS'S SIGNATURE | NAME | DATE |
| | | |
| Nurse / counsellors signature | Name | Date |
| | | |

**Note: 3 copies: 1 for patient, 1 for researcher, 1 to be kept with hospital notes**

## 2.2 CONSENT FOR STORAGE & FUTURE TESTING OF STORED BLOOD SAMPLES

## PATIENT INFORMATION

***Please take time to read the following information carefully and discuss it with others if you wish. Ask us about anything that is not clear or if you would like more information. Thank you for reading this.***

*You have agreed to be screened for entry into an HIV treatment study of the Medical Research Council / Uganda Virus Research Institute. You were informed that some blood would be drawn from you for the diagnosis of health conditions affecting you.*

*Some of this blood will be stored and used for research* ***in the future*** *to answer questions related to HIV and increase the information we have about HIV in this community.*

We would therefore like to ask your permission for us to store some of your blood samples for use in research at a future date. This will not necessitate taking any extra blood from you over and above that necessary for the screening tests to be done.

Your stored blood will be used for studies related to HIV and other infections only. Studies may be performed on your stored blood to confirm some test results and if new methods of testing become available, the samples may be used to see if these new tests give the same results. No laboratory testing on human genetic material will be conducted on your stored blood.

Your samples will not be sold. No studies on your samples will be done without permission of an ethics committee, which watches over the safety and rights of research participants and must approve any research studies using your samples.

Because we do not yet know what specific tests will be done on the stored blood, and because these tests will be performed at some date in the future which is not yet determined, you will not be given the results from these tests. However, you can be assured that any results showing changes that could affect your disease or the outcome of your treatment will be made available to you and your doctors.

All information that is collected about you during the COSTOP study and all information that will become available during any future tests will be kept strictly confidential. Your samples are identified only by your Study number and names will never be used.

If however, you do not wish any of your blood sample to be stored for future research but still wish to be screened for entry into the COSTOP study, you are free to do so.

## CONSENT FOR STORAGE OF BLOOD SAMPLES & FUTURE TESTING ON STORED SAMPLES (COSTOP STUDY)

| | Please initial box if you agree |
|---|---|
| I have read or had someone read to me the information on stored blood samples and I have had an opportunity to ask questions about and discuss these tests | ☐ |
| I understand I may not receive the results of these tests | ☐ |
| I agree that blood samples taken during the screening for the COSTOP Study man be stored and used for future research related to HIV and other infectious diseases | ☐ |
| I do not agree that blood samples taken during screening for the COSTOP Study may be stored and used for future research related to HIV and other infectious diseases | ☐ |

______________________________________________

Name of patient Date Signature

Study Number:______________________

______________________________________________

Name of researcher Date Signature

NB: **3 copies: 1 for patient, 1 for researcher, 1 to be kept with clinic notes**

## 2.3 CONSENT FOR ENROLMENT

Centre: Entebbe ❍ Masaka ❍

| | | | | | | | |
|---|---|---|---|---|---|---|---|
| Date of enrolment | ☐☐ | ☐☐ | 2 0 ☐☐ | Clinic / hospital number | ☐☐☐☐☐☐☐☐☐☐ | | |
| Date of birth | ☐☐ | ☐☐ | 1 9 ☐☐ | Sex: | Male ☐ | Female ☐ | |

I have read /been read the information sheet concerning the COSTOP study and understand what will be required of me if I take part in the study. ☐

My questions concerning this study have been answered by:

.....................................................................................................

I understand that at any time, I may withdrawal from this study without giving a reason and without affecting my normal care and management. ☐

I understand that I will have to continue taking my antiretroviral drugs plus either cotrimoxazole (septrin) or placebo during the study. ☐

I am willing to allow access to my medical notes to check that the trial is being carried out correctly but understand that strict confidentiality will be maintained. ☐

I voluntarily agree to take part in the study. ☐

| Individuals signature / thumb print | Name | Date |
|---|---|---|
| | | |

| WITNESS'S SIGNATURE | NAME | DATE |
|---|---|---|
| | | |

| Nurse / counsellors signature | Name | Date |
|---|---|---|
| | | |

**NB: 3 copies: 1 for patient, 1 for researcher, 1 to be kept with hospital notes**

# APPENDIX 3: TOXICITY TABLE

Adapted from : DIVISION OF AIDS TABLE FOR GRADING THE SEVERITY OF ADULT AND PEDIATRIC ADVERSE EVENTS VERSION 1.0, DECEMBER, 2004; CLARIFICATION AUGUST 2009

| CLINICAL | | | | |
|---|---|---|---|---|
| PARAMETER | GRADE 1<br>MILD | GRADE 2<br>MODERATE | GRADE 3<br>SEVERE | GRADE 4<br>POTENTIALLY LIFE-THREATENING |
| **ESTIMATING SEVERITY GRADE** | | | | |
| **Clinical adverse event NOT identified elsewhere in this DAIDS AE Grading Table** | Symptoms causing no or minimal interference with usual social & functional activities | Symptoms causing greater than minimal interference with usual social & functional activities | Symptoms causing inability to perform usual social & functional activities | Symptoms causing inability to perform basic self-care functions OR Medical or operative intervention indicated to prevent permanent impairment, persistent disability, or death |
| **SYSTEMIC** | | | | |
| **Acute systemic allergic reaction** | Localized urticaria (wheals) with no medical intervention indicated | Localized urticaria with medical intervention indicated OR Mild angioedema with no medical intervention indicated | Generalized urticaria OR Angioedema with medical intervention indicated OR Symptomatic mild bronchospasm | Acute anaphylaxis OR Life-threatening bronchospasm OR laryngeal edema |
| **Chills** | Symptoms causing no or minimal interference with usual social & functional activities | Symptoms causing greater than minimal interference with usual social & functional activities | Symptoms causing inability to perform usual social & functional activities | NA |
| **Fatigue**<br>**Malaise** | Symptoms causing no or minimal interference with usual social & functional activities | Symptoms causing greater than minimal interference with usual social & functional activities | Symptoms causing inability to perform usual social & functional activities | Incapacitating fatigue/ malaise symptoms causing inability to perform basic self-care functions |
| **Fever (nonaxillary)** | 37.7 – 38.6°C | 38.7 – 39.3°C | 39.4 – 40.5°C | > 40.5°C |
| **Pain (indicate body site)**<br>**DO NOT use for pain due to injection (See Injection Site Reactions: Injection site pain)**<br>**See also Headache, Arthralgia, and Myalgia** | Pain causing no or minimal interference with usual social & functional activities | Pain causing greater than minimal interference with usual social & functional activities | Pain causing inability to perform usual social & functional activities | Disabling pain causing inability to perform basic self-care functions OR Hospitalization (other than emergency room visit) indicated |

| | | | | |
|---|---|---|---|---|
| **Unintentional weight loss** | NA | 5 – 9% loss in body weight from baseline | 10 – 19% loss in body weight from baseline | ≥ 20% loss in body weight from baseline OR Aggressive intervention indicated [e.g., tube feeding or total parenteral nutrition (TPN)] |
| **INFECTION** | | | | |
| **Infection (any other than HIV infection)** | Localized, no systemic antimicrobial treatment indicated AND Symptoms causing no or minimal interference with usual social & functional activities | Systemic antimicrobial treatment indicated OR Symptoms causing greater than minimal interference with usual social & functional activities | Systemic antimicrobial treatment indicated AND Symptoms causing inability to perform usual social & functional activities OR Operative intervention (other than simple incision and drainage) indicated | Life-threatening consequences (e.g., septic shock) |
| **INJECTION SITE REACTIONS** | | | | |
| **Injection site pain (pain without touching)**<br>**Or**<br>**Tenderness (pain when area is touched)** | Pain/tenderness causing no or minimal limitation of use of limb | Pain/tenderness limiting use of limb OR Pain/tenderness causing greater than minimal interference with usual social & functional activities | Pain/tenderness causing inability to perform usual social & functional activities | Pain/tenderness causing inability to perform basic self-care function OR Hospitalization (other than emergency room visit) indicated for management of pain/tenderness |
| **Localised Injection site Reaction** | **Erythema OR Induration of 5x5 cm – 9x9 cm (or 25 $cm^2$ – 81$cm^2$)** | Erythema OR Induration OR Edema > 9 cm any diameter (or > 81 $cm^2$) | Ulceration OR Secondary infection OR Phlebitis OR Sterile abscess OR Drainage | Necrosis (involving dermis and deeper tissue) |
| **Pruritis associated with injection**<br>**See also Skin: Pruritis (itching - no skin lesions)** | Itching localized to injection site AND Relieved spontaneously or with < 48 hours treatment | Itching beyond the injection site but not generalized OR Itching localized to injection site requiring ≥ 48 hours treatment | Generalized itching causing inability to perform usual social & functional activities | NA |
| **SKIN – DERMATOLOGICAL** | | | | |
| **Alopecia** | Thinning detectable by study participant (or by caregiver for young children and disabled adults) | Thinning or patchy hair loss detectable by health care provider | Complete hair loss | NA |

| | | | | |
|---|---|---|---|---|
| **Cutaneous reaction – rash** | Localized macular rash | Diffuse macular, maculopapular, or morbilliform rash OR Target lesions | Diffuse macular, maculopapular, or morbilliform rash with vesicles or limited number of bullae OR Superficial ulcerations of mucous membrane limited to one site | Extensive or generalized bullous lesions OR Stevens-Johnson syndrome OR Ulceration of mucous membrane involving two or more distinct mucosal sites OR Toxic epidermal necrolysis (TEN) |
| **Hyperpigmentation** | Slight or localized | Marked or generalized | NA | NA |
| **Hypopigmentation** | Slight or localized | Marked or generalized | NA | NA |
| **Pruritis (itching – no skin lesions)** **(See also Injection Site Reactions: Pruritis associated with injection)** | Itching causing no or minimal interference with usual social & functional activities | Itching causing greater than minimal interference with usual social & functional activities | Itching causing inability to perform usual social & functional activities | NA |
| **CARDIOVASCULAR** | | | | |
| **Cardiac arrhythmia (general)** **(By ECG or physical exam)** | **Asymptomatic AND No intervention indicated** | Asymptomatic AND Non-urgent medical intervention indicated | Symptomatic, non-life-threatening AND Non-urgent medical intervention indicated | Life-threatening arrhythmia OR Urgent intervention indicated |
| **Cardiac-ischemia/infarction** | NA | NA | Symptomatic ischemia (stable angina) OR Testing consistent with ischemia | Unstable angina OR Acute myocardial infarction |
| **Hemorrhage (significant acute blood loss)** | NA | Symptomatic AND No transfusion indicated | Symptomatic AND Transfusion of ≤ 2 units packed RBCs (for children ≤ 10 cc/kg) indicated | Life-threatening hypotension OR Transfusion of > 2 units packed RBCs (for children > 10 cc/kg) indicated |
| **Hypertension (with repeat testing at same visit)** | **140 – 159 mmHg systolic OR 90 – 99 mmHg diastolic** | 160 – 179 mmHg systolic OR 100 – 109 mmHg diastolic | ≥ 180 mmHg systolic OR ≥ 110 mmHg diastolic | Life-threatening consequences (e.g., malignant hypertension) OR Hospitalization indicated (other than emergency room visit) |
| **Hypotension** | NA | Symptomatic, corrected with oral fluid replacement | Symptomatic, IV fluids indicated | Shock requiring use of vasopressors or mechanical assistance to maintain blood pressure |

| | | | | |
|---|---|---|---|---|
| **Pericardial effusion** | Asymptomatic, small effusion requiring no intervention | Asymptomatic, moderate or larger effusion requiring no intervention | Effusion with non-life threatening physiologic consequences OR Effusion with non-urgent intervention indicated | Life-threatening consequences (e.g., tamponade) OR Urgent intervention indicated |
| **Prolonged PR interval** | PR interval 0.21 – 0.25 sec | PR interval > 0.25 sec | Type II 2$^{nd}$ degree AV block OR Ventricular pause > 3.0 sec | Complete AV block |
| **Prolonged QTc** | **Asymptomatic, QTc interval 0.45 – 0.47 sec OR Increase interval < 0.03 sec above baseline** | Asymptomatic, QTc interval 0.48 – 0.49 sec OR Increase in interval 0.03 – 0.05 sec above baseline | Asymptomatic, QTc interval ≥ 0.50 sec OR Increase in interval ≥ 0.06 sec above baseline | Life-threatening consequences, e.g. Torsade de pointes or other associated serious ventricular dysrhythmia |
| **Thrombosis/embolism** | NA | Deep vein thrombosis AND No intervention indicated (e.g., anticoagulation, lysis filter, invasive procedure) | Deep vein thrombosis AND Intervention indicated (e.g., anticoagulation, lysis filter, invasive procedure) | Embolic event (e.g., pulmonary embolism, life-threatening thrombus) |
| **Vasovagal episode (associated with a procedure of any kind)** | Present without loss of consciousness | Present with transient loss of consciousness | NA | NA |
| **Ventricular dysfunction (congestive heart failure)** | NA | Asymptomatic diagnostic finding AND intervention indicated | New onset with symptoms OR Worsening symptomatic congestive heart failure | Life-threatening congestive heart failure |
| **GASTROINTESTINAL** | | | | |
| **Anorexia** | Loss of appetite without decreased oral intake | Loss of appetite associated with decreased oral intake without significant weight loss | Loss of appetite associated with significant weight loss | Life-threatening consequences OR Aggressive intervention indicated [e.g., tube feeding or total parenteral nutrition (TPN)] |
| **Ascites** | Asymptomatic | Symptomatic AND Intervention indicated (e.g., diuretics or therapeutic paracentesis) | Symptomatic despite intervention | Life-threatening consequences |
| **Cholecystitis** | NA | Symptomatic AND Medical intervention indicated | Radiologic, endoscopic, or operative intervention indicated | Life-threatening consequences (e.g., sepsis or perforation) |

| | | | | |
|---|---|---|---|---|
| **Constipation** | NA | Persistent constipation requiring regular use of dietary modifications, laxatives, or enemas | Obstipation with manual evacuation indicated | Life-threatening consequences (e.g., obstruction) |
| **Diarrhea** | **Transient or intermittent episodes of unformed stools OR Increase of ≤ 3 stools over baseline per 24-hour period** | Persistent episodes of unformed to watery stools OR Increase of 4 – 6 stools over baseline per 24-hour period | Bloody diarrhea OR Increase of ≥ 7 stools per 24-hour period OR IV fluid replacement indicated | Life-threatening consequences (e.g., hypotensive shock) |
| **Dysphagia-Odynophagia** | Symptomatic but able to eat usual diet | Symptoms causing altered dietary intake without medical intervention indicated | Symptoms causing severely altered dietary intake with medical intervention indicated | Life-threatening reduction in oral intake |
| **Mucositis/stomatitis (clinical exam)** **Indicate site (e.g., larynx, oral)** **See Genitourinary for Vulvovaginitis** **See also Dysphagia-Odynophagia and Proctitis** | Erythema of the mucosa | Patchy pseudomembranes or ulcerations | Confluent pseudomembranes or ulcerations OR Mucosal bleeding with minor trauma | Tissue necrosis OR Diffuse spontaneous mucosal bleeding OR Life-threatening consequences (e.g., aspiration, choking) |
| **Nausea** | Transient (< 24 hours) or intermittent nausea with no or minimal interference with oral intake | Persistent nausea resulting in decreased oral intake for 24 – 48 hours | Persistent nausea resulting in minimal oral intake for > 48 hours OR Aggressive rehydration indicated (e.g., IV fluids) | Life-threatening consequences (e.g., hypotensive shock) |
| **Pancreatitis** | NA | Symptomatic AND Hospitalization not indicated (other than emergency room visit) | Symptomatic AND Hospitalization indicated (other than emergency room visit) | Life-threatening consequences (e.g., circulatory failure, hemorrhage, sepsis) |
| **Proctitis (functional-symptomatic)** **Also see Mucositis/stomatitis for clinical exam** | Rectal discomfort AND No intervention indicated | Symptoms causing greater than minimal interference with usual social & functional activities OR Medical intervention indicated | Symptoms causing inability to perform usual social & functional activities OR Operative intervention indicated | Life-threatening consequences (e.g., perforation) |
| **Vomiting** | Transient or intermittent vomiting with no or minimal interference with oral intake | Frequent episodes of vomiting with no or mild dehydration | Persistent vomiting resulting in orthostatic hypotension OR Aggressive rehydration indicated (e.g., IV fluids) | Life-threatening consequences (e.g., hypotensive shock) |

| NEUROLOGIC | | | | |
|---|---|---|---|---|
| **Alteration in personality-behavior or in mood (e.g., agitation, anxiety, depression, mania, psychosis)** | Alteration causing no or minimal interference with usual social & functional activities | Alteration causing greater than minimal interference with usual social & functional activities | Alteration causing inability to perform usual social & functional activities | Behavior potentially harmful to self or others (e.g., suicidal and homicidal ideation or attempt, acute psychosis) OR Causing inability to perform basic self-care functions |
| **Altered Mental Status**<br>**For Dementia, see Cognitive and behavioral/attentional disturbance (including dementia and attention deficit disorder)** | Changes causing no or minimal interference with usual social & functional activities | Mild lethargy or somnolence causing greater than minimal interference with usual social & functional activities | Confusion, memory impairment, lethargy, or somnolence causing inability to perform usual social & functional activities | Delirium OR obtundation, OR coma |
| **Ataxia** | Asymptomatic ataxia detectable on exam OR Minimal ataxia causing no or minimal interference with usual social & functional activities | Symptomatic ataxia causing greater than minimal interference with usual social & functional activities | Symptomatic ataxia causing inability to perform usual social & functional activities | Disabling ataxia causing inability to perform basic self-care functions |
| **Cognitive and behavioral/attentional disturbance (including dementia and attention deficit disorder)** | Disability causing no or minimal interference with usual social & functional activities OR Specialized resources not indicated | Disability causing greater than minimal interference with usual social & functional activities OR Specialized resources on part-time basis indicated | Disability causing inability to perform usual social & functional activities OR Specialized resources on a full-time basis indicated | Disability causing inability to perform basic self-care functions OR Institutionalization indicated |
| **CNS ischemia (acute)** | NA | NA | Transient ischemic attack | Cerebral vascular accident (CVA, stroke) with neurological deficit |
| **Headache** | Symptoms causing no or minimal interference with usual social & functional activities | Symptoms causing greater than minimal interference with usual social & functional activities | Symptoms causing inability to perform usual social & functional activities | Symptoms causing inability to perform basic self-care functions OR Hospitalization indicated (other than emergency room visit) OR Headache with significant impairment of alertness or other neurologic function |

| | | | | |
|---|---|---|---|---|
| **Insomnia** | NA | Difficulty sleeping causing greater than minimal interference with usual social & functional activities | Difficulty sleeping causing inability to perform usual social & functional activities | Disabling insomnia causing inability to perform basic self-care functions |
| **Neuromuscular weakness (including myopathy & neuropathy)** | Asymptomatic with decreased strength on exam OR Minimal muscle weakness causing no or minimal interference with usual social & functional activities | Muscle weakness causing greater than minimal interference with usual social & functional activities | Muscle weakness causing inability to perform usual social & functional activities | Disabling muscle weakness causing inability to perform basic self-care functions OR Respiratory muscle weakness impairing ventilation |
| **Neurosensory alteration (including paresthesia and painful neuropathy)** | Asymptomatic with sensory alteration on exam or minimal paresthesia causing no or minimal interference with usual social & functional activities | Sensory alteration or paresthesia causing greater than minimal interference with usual social & functional activities | Sensory alteration or paresthesia causing inability to perform usual social & functional activities | Disabling sensory alteration or paresthesia causing inability to perform basic self-care functions |
| **Seizure: (new onset)**<br>**See also Seizure: (known pre-existing seizure disorder)** | NA | 1 seizure | 2 – 4 seizures | Seizures of any kind which are prolonged, repetitive (e.g., status epilepticus), or difficult to control (e.g., refractory epilepsy) |
| **Seizure: (known pre-existing seizure disorder)**<br>**For worsening of existing epilepsy the grades should be based on an increase from previous level of control to any of these levels.** | NA | Increased frequency of pre-existing seizures (non-repetitive) without change in seizure character OR Infrequent break-through seizures while on stable medication in a previously controlled seizure disorder | Change in seizure character from baseline either in duration or quality (e.g., severity or focality) | Seizures of any kind which are prolonged, repetitive (e.g., status epilepticus), or difficult to control (e.g., refractory epilepsy) |
| **Syncope (not associated with a procedure)** | NA | Present | NA | NA |
| **Vertigo** | Vertigo causing no or minimal interference with usual social & functional activities | Vertigo causing greater than minimal interference with usual social & functional activities | Vertigo causing inability to perform usual social & functional activities | Disabling vertigo causing inability to perform basic self-care functions |

| | | | | |
|---|---|---|---|---|
| **RESPIRATORY** | | | | |
| **Bronchospasm (acute)** | FEV1 or peak flow reduced to 70 – 80% | FEV1 or peak flow 50 – 69% | FEV1 or peak flow 25 – 49% | Cyanosis OR FEV1 or peak flow < 25% OR Intubation |
| **Dyspnoea or respiratory distress** | **Dyspnea on exertion with no or minimal interference with usual social & functional activities** | Dyspnea on exertion causing greater than minimal interference with usual social & functional activities | Dyspnea at rest causing inability to perform usual social & functional activities | Respiratory failure with ventilatory support indicated |
| **MUSCULOSKELETAL** | | | | |
| **Arthralgia**<br>**See also Arthritis** | Joint pain causing no or minimal interference with usual social & functional activities | Joint pain causing greater than minimal interference with usual social & functional activities | Joint pain causing inability to perform usual social & functional activities | Disabling joint pain causing inability to perform basic self-care functions |
| **Arthritis**<br>**See also Arthralgia** | Stiffness or joint swelling causing no or minimal interference with usual social & functional activities | Stiffness or joint swelling causing greater than minimal interference with usual social & functional activities | Stiffness or joint swelling causing inability to perform usual social & functional activities | Disabling joint stiffness or swelling causing inability to perform basic self-care functions |
| **Bone Mineral Loss** | BMD t-score -2.5 to -1.0 | BMD t-score < -2.5 | Pathological fracture (including loss of vertebral height) | Pathologic fracture causing life-threatening consequences |
| **Myalgia**<br>**(non-injection site)** | Muscle pain causing no or minimal interference with usual social & functional activities | Muscle pain causing greater than minimal interference with usual social & functional activities | Muscle pain causing inability to perform usual social & functional activities | Disabling muscle pain causing inability to perform basic self-care functions |
| **Osteonecrosis** | NA | Asymptomatic with radiographic findings AND No operative intervention indicated | Symptomatic bone pain with radiographic findings OR Operative intervention indicated | Disabling bone pain with radiographic findings causing inability to perform basic self-care functions |
| **GENITOURINARY** | | | | |
| **Cervicitis (symptoms)**<br>**(For use in studies evaluating topical study agents)**<br>**For other cervicitis see Infection: Infection (any other than HIV infection)** | Symptoms causing no or minimal interference with usual social & functional activities | Symptoms causing greater than minimal interference with usual social & functional activities | Symptoms causing inability to perform usual social & functional activities | Symptoms causing inability to perform basic self-care functions |

| | | | | |
|---|---|---|---|---|
| **Cervicitis (clinical exam)** **(For use in studies evaluating topical study agents)** **For other cervicitis see Infection: Infection (any other than HIV infection)** | Minimal cervical abnormalities on examination (erythema, mucopurulent discharge, or friability) OR Epithelial disruption < 25% of total surface | Moderate cervical abnormalities on examination (erythema, mucopurulent discharge, or friability) OR Epithelial disruption of 25 – 49% total surface | Severe cervical abnormalities on examination (erythema, mucopurulent discharge, or friability) OR Epithelial disruption 50 – 75% total surface | Epithelial disruption > 75% total surface |
| **Inter-menstrual bleeding (IMB)** | Spotting observed by participant OR Minimal blood observed during clinical or colposcopic examination | Inter-menstrual bleeding not greater in duration or amount than usual menstrual cycle | Inter-menstrual bleeding greater in duration or amount than usual menstrual cycle | Hemorrhage with life-threatening hypotension OR Operative intervention indicated |
| **Urinary tract obstruction (e.g., stone)** | NA | Signs or symptoms of urinary tract obstruction without hydronephrosis or renal dysfunction | Signs or symptoms of urinary tract obstruction with hydronephrosis or renal dysfunction | Obstruction causing life-threatening consequences |
| **Vulvovaginitis (symptoms)** **(Use in studies evaluating topical study agents)** **For other vulvovaginitis see Infection: Infection (any other than HIV infection)** | Symptoms causing no or minimal interference with usual social & functional activities | Symptoms causing greater than minimal interference with usual social & functional activities | Symptoms causing inability to perform usual social & functional activities | Symptoms causing inability to perform basic self-care functions |
| **Vulvovaginitis (clinical exam)** **(Use in studies evaluating topical study agents)** **For other vulvovaginitis see Infection: Infection (any other than HIV infection)** | Minimal vaginal abnormalities on examination OR Epithelial disruption < 25% of total surface | Moderate vaginal abnormalities on examination OR Epithelial disruption of 25 - 49% total surface | Severe vaginal abnormalities on examination OR Epithelial disruption 50 - 75% total surface | Vaginal perforation OR Epithelial disruption > 75% total surface |
| **OCULAR/VISUAL** | | | | |
| **Uveitis** | Asymptomatic but detectable on exam | Symptomatic anterior uveitis OR Medical intervention indicated | Posterior or pan-uveitis OR Operative intervention indicated | Disabling visual loss in affected eye(s) |

| | | | | |
|---|---|---|---|---|
| **Visual changes (from baseline)** | Visual changes causing no or minimal interference with usual social & functional activities | Visual changes causing greater than minimal interference with usual social & functional activities | Visual changes causing inability to perform usual social & functional activities | Disabling visual loss in affected eye(s) |
| **ENDOCRINE/METABOLIC** | | | | |
| **Abnormal fat accumulation (e.g., back of neck, breasts, abdomen)** | Detectable by study participant (or by caregiver for young children and disabled adults) | Detectable on physical exam by health care provider | Disfiguring OR Obvious changes on casual visual inspection | NA |
| **Diabetes mellitus** | NA | New onset without need to initiate medication OR Modification of current medications to regain glucose control | New onset with initiation of medication indicated OR Diabetes uncontrolled despite treatment modification | Life-threatening consequences (e.g., ketoacidosis, hyperosmolar non-ketotic coma) |
| **Gynecomastia** | Detectable by study participant or caregiver (for young children and disabled adults) | Detectable on physical exam by health care provider | Disfiguring OR Obvious on casual visual inspection | NA |
| **Hyperthyroidism** | Asymptomatic | Symptomatic causing greater than minimal interference with usual social & functional activities OR Thyroid suppression therapy indicated | Symptoms causing inability to perform usual social & functional activities OR Uncontrolled despite treatment modification | Life-threatening consequences (e.g., thyroid storm) |
| **Hypothyroidism** | Asymptomatic | Symptomatic causing greater than minimal interference with usual social & functional activities OR Thyroid replacement therapy indicated | Symptoms causing inability to perform usual social & functional activities OR Uncontrolled despite treatment modification | Life-threatening consequences (e.g., myxedema coma) |
| **Lipoatrophy (e.g., fat loss from the face, extremities, buttocks)** | Detectable by study participant (or by caregiver for young children and disabled adults) | Detectable on physical exam by health care provider | Disfiguring OR Obvious on casual visual inspection | NA |

# APPENDIX 4: CLINICAL EVENT CRITERIA

## Clinical Criteria for HIV-Related WHO Clinical Events.

Source: Revised WHO Clinical Staging and Immunological Classification of HIV and case definition of HIV for surveillance, May 2006.

| Clinical event | Clinical diagnosis | Definitive diagnosis |
|---|---|---|
| **Clinical Stage 1** | | |
| Asymptomatic | No HIV related symptoms reported and no signs on examination. | Not applicable |
| Persistent generalized lymphadenopathy (PGL) | Painless enlarged lymph nodes >1 cm, in two or more non-contiguous sites (excluding inguinal), in absence of known cause & persisting for ≥3 months | Histology |
| **Clinical Stage 2** | | |
| Moderate unexplained weight loss (<10% of body weight) | Reported unexplained weight loss. In pregnancy failure to gain weight. | Documented weight loss <10% of body weight. |
| Recurrent bacterial upper respiratory tract infections (current event plus one or more in last six-month period) | Symptom complex, e.g. unilateral face pain with nasal discharge (sinusitis), painful inflamed eardrum (otitis media), or tonsillo-pharyngitis without features of viral infection (e.g. coryza, cough). | Laboratory studies where available, e.g. culture of suitable body fluid. |
| Herpes zoster | Painful vesicular rash in dermatomal distribution of a nerve supply does not cross midline. | Clinical diagnosis |
| Angular cheilitis | Splits or cracks at the angle of the mouth not due to iron or vitamin deficiency, and usually respond to antifungal treatment. | Clinical diagnosis. |
| Recurrent oral ulcerations (two or more episodes in last six months) | Aphthous ulceration, typically painful with a halo of inflammation and a yellow-grey pseudomembrane. | Clinical diagnosis. |
| Papular pruritic eruption | Papular pruritic lesions, often with marked post-inflammatory pigmentation. | Clinical diagnosis. |
| Seborrhoeic dermatitis | Itchy scaly skin condition, particularly affecting hairy areas (scalp, axillae, upper trunk and groin). | Clinical diagnosis. |
| Fungal nail infections | Paronychia (painful red and swollen nail bed) or onycholysis (separation of the nail from the nail bed) of the fingernails (white discolouration - especially involving proximal part of nail plate – with thickening & separation of nail from nail bed). | Fungal culture of nail/nail plate material. |
| **Clinical Stage 3** | | |
| Severe unexplained weight loss (more than 10% of body weight) | Reported unexplained weight loss (>10% of body weight) and visible thinning of face, waist and extremities with obvious wasting or body mass index <18.5. In pregnancy weight loss may be masked. | Documented loss of more than 10% of body weight. |
| Unexplained chronic diarrhoea for longer than one month | Chronic diarrhoea (loose or watery stools three or more times daily) reported for longer than one month. | Not required but confirmed if three or more stools observed and documented as unformed, and two or more stool tests reveal no pathogens |
| Unexplained persistent fever (intermittent or constant and lasting for longer than one month) | Reports of fever or night sweats for more than one month, either intermittent or constant with reported lack of response to antibiotics or antimalarials, without other obvious foci of disease reported or found on examination. Malaria must be excluded in malarious areas. | Documented fever >37.6 °C. with negative blood culture, negative Ziehl-Nielsen (ZN) stain, negative malaria slide, normal or unchanged chest X-ray (CXR) and no other obvious focus of infection. |

| | | |
|---|---|---|
| Oral candidiasis | Persistent or recurring creamy white curd-like plaques which can be scraped off (pseudomembranous), or red patches on tongue, palate or lining of mouth, usually painful or tender (erythematous form) | Clinical diagnosis |
| Oral hairy leukoplakia | Fine white small linear or corrugated lesions on lateral borders of the tongue, which do not scrape off. | Clinical diagnosis |
| Pulmonary TB (current) | Chronic symptoms: (lasting ≥2-3 weeks) cough, haemoptysis, shortness of breath, chest pain, weight loss, fever, night sweats, PLUS either positive sputum smear<br>OR<br>Negative sputum smear AND compatible chest radiograph (including but not restricted to upper lobe infiltrates, cavitation, pulmonary fibrosis and shrinkage). No evidence of extrapulmonary disease | Isolation of *M. tuberculosis* on sputum culture or histology of lung biopsy (together with compatible symptoms). |
| Severe bacterial infection (e.g. pneumonia, meningitis, empyema, pyomyositis, bone or joint infection, bacteraemia, severe pelvic inflammatory disease ) | Fever accompanied by specific symptoms or signs that localize infection, and response to appropriate antibiotic. | Isolation of bacteria from appropriate clinical specimens (i.e. usually sterile sites). |
| Acute necrotizing ulcerative gingivitis or necrotizing ulcerative periodontitis | Severe pain, ulcerated gingival papillae, loosening of teeth, spontaneous bleeding, bad odour, and rapid loss of bone and/or soft tissue. | Clinical diagnosis. |
| Unexplained anaemia (<8g/dl ), neutropenia (<0.5 $\times 10^9$/L or chronic (more than one month) thrombocytopenia(<50 $\times 10^9$/L) | Not presumptive clinical diagnosis. | Diagnosed on laboratory testing and not explained by other non-HIV conditions. Not responding to standard therapy with haematinics, antimalarials or anthelmintics as outlined in relevant national treatment guidelines, WHO IMCI guidelines or other relevant guidelines. |
| Clinical Stage 4 | | |
| HIV wasting syndrome | Reported unexplained weight loss (>10% body weight), with obvious wasting or body mass index <18.5.<br>PLUS EITHER<br>unexplained chronic diarrhoea (loose or watery stools three or more times daily) reported for longer than one month.<br>OR<br>Reports of fever or night sweats for more than one month without other cause and lack of response to antibiotics or antimalarials. Malaria must be excluded in malarious areas. | Documented weight loss >10% of body weight;<br>plus<br>two or more unformed stools negative for pathogens<br>or<br>Documented temperature of > 37.6 °C or more with no other cause of disease, negative blood culture, negative malaria slide and normal or unchanged CXR. |
| *Pneumocystis* pneumonia | Dyspnoea on exertion or nonproductive cough of recent onset (within the past 3 months), tachypnoea and fever; AND Chest x-ray evidence of diffuse bilateral interstitial infiltrates AND No evidence of a bacterial pneumonia. Bilateral crepitations on auscultation with or without reduced air entry. | Cytology or immunofluorescent microscopy of induced sputum or bronchoalveolar lavage (BAL), or histology of lung tissue. |
| Recurrent bacterial pneumonia (this episode plus one or more episodes in last 6 months) | Current episode plus one or more previous episodes in last 6 months .Acute onset (<2 weeks) of symptoms (e.g. fever, cough, dyspnoea, and chest pain) PLUS new consolidation on clinical examination or CXR. Response to antibiotics. | Positive culture or antigen test of a compatible organism. |

| | | |
|---|---|---|
| Chronic herpes simplex virus (HSV) infection (orolabial, genital or anorectal) of more than one month, or visceral of any duration | Painful, progressive anogenital or orolabial ulceration; lesions caused by recurrent HSV infection and reported for more than one month. History of previous episodes. Visceral HSV requires definitive diagnosis. | Positive culture or DNA (by PCR) of HSV or compatible cytology/histology. |
| Oesophageal candidiasis | Recent onset of retrosternal pain or difficulty on swallowing (food and fluids) together with oral candidiasis. | Macroscopic appearance at endoscopy or bronchoscopy, or by microscopy/histology. |
| Extrapulmonary TB | Systemic illness (e.g. fever, night sweats, weakness and weight loss). Other evidence for extrapulmonary or disseminated TB varies by site: Pleural, pericardial, peritoneal involvement, meningitis, mediastinal or abdominal lymphadenopathy, osteitis.<br><br>Miliary TB diffuse uniformly distributed small miliary shadows or micronodules on CXR.<br><br>Discrete cervical lymph node *M. tuberculosis* infection is usually considered a less severe form of extra pulmonary tuberculosis. | *M. tuberculosis* isolation or compatible histology from appropriate site, together with compatible symptoms/signs (if culture/histology is from respiratory specimen then must other have evidence of extra pulmonary disease). |
| Kaposi's sarcoma | Typical appearance in skin or oropharynx of persistent, initially flat, patches with a pink or blood-bruise colour, skin lesions that usually develop into violaceous plaques or nodules. | Macroscopic appearance at endoscopy or bronchoscopy, or by histology. |
| CMV disease (other than liver, spleen or lymph node). | Retinitis only: may be diagnosed by experienced clinicians. Typical eye lesions on fundoscopic examination: discrete patches of retinal whitening with distinct borders, spreading centrifugally, often following blood vessels, associated with retinal vasculitis, haemorrhage and necrosis. | Compatible histology or CMV demonstrated in CSF by culture or DNA (by PCR). |
| CNS toxoplasmosis | Recent onset of a focal neurological abnormality or reduced level of consciousness AND response within 10 days to specific therapy. | Positive serum toxoplasma antibody AND (if available)single/multiple intracranial mass lesion on neuro-imaging (CT or MRI) |
| HIV encephalopathy | Clinical finding of disabling cognitive and/or motor dysfunction interfering with activities of daily living, progressing over weeks or months in the absence of a concurrent illness or condition other than HIV infection which might explain the findings. | Diagnosis of exclusion: and (if available) neuro-imaging (CT or MRI) |
| Extrapulmonary cryptococcosis (including meningitis) | Meningitis: usually sub acute, fever with increasing severe headache, meningism, confusion, behavioural changes that responds to cryptococcal therapy. | Isolation of *Cryptococcus neoformans* from extrapulmonary site or positive cryptococcal antigen test (CRAG) on CSF/blood. |
| Disseminated non-tuberculous mycobacteria infection | No presumptive clinical diagnosis. | Diagnosed by finding atypical mycobacterial species from stool, blood, body fluid or other body tissue, excluding lung. |

| | | |
|---|---|---|
| Progressive multi focal leukoencephalopathy (PML) | No presumptive clinical diagnosis | Progressive neurological disorder (cognitive dysfunction, gait/speech disorder, visual loss, limb weakness and cranial nerve palsies) together with hypodense white matter lesions on neuro-imaging or positive polyomavirus JC (JCV) PCR on CSF. |
| Cryptosporidiosis (with diarrhoea lasting more than one month) | No presumptive clinical diagnosis. | Cysts identified on modified ZN microscopic examination of unformed stool. |
| Chronic isosporiasis | No presumptive clinical diagnosis. | Identification of *Isospora* |
| Disseminated mycosis (coccidiomycosis, histoplasmosis) | No presumptive clinical diagnosis. | Histology, antigen detection or culture from clinical specimen or blood culture. |
| Recurrent non-typhoid salmonella bacteraemia | No presumptive clinical diagnosis. | Blood culture. |
| Lymphoma (cerebral or B cell non-Hodgkin) or other solid HIV associated tumours. | No presumptive clinical diagnosis | Histology of relevant specimen or for CNS tumours neuroimaging techniques |
| Invasive cervical carcinoma | No presumptive clinical diagnosis. | Histology or cytology. |
| Visceral leishmaniasis | No presumptive clinical diagnosis. | Diagnosed by histology (amastigotes visualized) or culture from any appropriate clinical specimen. |
| HIV-associated nephropathy | No presumptive clinical diagnosis | Renal biopsy |
| HIV-associated cardiomyopathy | No presumptive clinical diagnosis | Cardiomegaly and evidence of poor left ventricular function confirmed by echocardiography. |

# APPENDIX 5: TOXICITY MANAGEMENT TABLES

## Symptom directed toxicity management- Co-trimoxazole hypersensitivity

Source: WHO Guidelines on co-trimoxazole prophylaxis for HIV-related infections among children, adolescents and adults in resource-limited settings. 2006

| Toxicity | Clinical Description | Recommendation |
|---|---|---|
| GRADE 1 | Erythema | Continue co-trimoxazole prophylaxis with careful and repeated observation and followup.<br><br>Provide symptomatic treatment, such as antihistamines, if available |
| GRADE 2 | Diffuse maculopapular rash, dry desquamation | Continue co-trimoxazole prophylaxis with careful and repeated observation and followup.<br><br>Provide symptomatic treatment, such as antihistamines, if available |
| GRADE 3 | Vesiculation, mucosal ulceration | Co-trimoxazole should be discontinued until the adverse effect has completely resolved (usually two weeks), and then reintroduction or desensitization can be considered |
| GRADE 4 | Exfoliative dermatitis, Stevens-Johnson syndrome or erythema multiforme, moist desquamation | Co-trimoxazole should be permanently discontinued |

## Symptom directed toxicity management – Antiretroviral Drugs

Source: WHO Guidelines: Antiretroviral Therapy for HIV Infection in Adults and Adolescents in Resource-Limited Settings. 2006

| AdverseEffect | Major ARVs | Recommendations |
|---|---|---|
| Acute pancreatitis | d4T and ddI | Discontinue ART. Give supportive treatment and laboratory monitoring. Resume ART with an NRTI with low pancreatic toxicity risk. AZT, ABC, TDF and 3TC are less likely to cause this type of toxicity. |
| Diarrhea | ddI (buffered formulation), NVF, LPV/r and SQV/r | Usually self-limited, without need to discontinue ART. Symptomatic treatment should be offered. |
| Drug eruptions (mild to severe, including Stevens-Johnson syndrome or toxic epidermal necrolysis) | NVP, EFV (rarely) | In very mild cases, antihistamines and strict observation; there may be regression without need to change ART. If mild/moderate rash, non-progressing and without mucosal involvement or systemic signs, consider a single NNRTI substitution (i.e., from NVP to EFV). In moderate and severe cases, discontinue ART and give supportive treatment. After resolution, resume ART with 3 NRTI or 2 NRTI + PI regimens. |
| Dyslipidemia, insulin resistance and hyperglycemia | PIs | Consider replacing the suspected PI by drugs with less risk of metabolic toxicity (e.g., NFV). Adequate diet , physical exercise and antilipemic drugs should be considered. |
| GI intolerance, with taste changes, nausea, vomiting, abdominal pain and diarrhea. | All ARVs (less frequent with d4T, 3TC, FTC and ABC) | Usually self-limited, without need to discontinue ART. Symptomatic treatment should be offered. |
| Hematological toxicities (particularly anemia and leucopenia) | AZT | If severe (Hg<6.5 g% and /or ANC <500 cells/mm$^3$) replace by an ARV with minimal or no bone marrow toxicity (e.g. d4T, ABC or TDF) and consider blood transfusion. |
| Hepatitis | All ARVs (particularly with NVP and ritonavir boosted PIs) | Intense elevations of ALT associated with clinical features were described with NVP; however, changes (of varying intensity) may be observed with all ARVs, mediated by different mechanisms.<br>If ALT >5-fold the basal level, discontinue ART and monitor. After resolution, replace the drug most likely associated. |
| Hyperbiliruubinemia (indirect) | ATV | Generally asymptomatic, but can cause scleral icterus (without ALT elevations). Replace ATV for other PI. |
| Hypersensitivity reaction with systemic respiratory,fever and without mucosal involvement. | ABC | Discontinue ABC and **do not restart**. Symptomatic treatment. Reexposure may lead to a severe and potentially life-threatening reaction. |
| Lactic acidosis | All NRTIs (particularly d4T and ddI) | Discontinue ART and give supportive treatment. After clinical resolution, resume ART, replacing the offending ITRN. ABC, TDF and 3TC are less likely to cause this type of toxicity. |
| Lipoatrophy and lipodystrophy | All NRTIs (particularly d4T) | Early replacement of the suspected ARV drug (e.g., d4T for TDF or ABC). Consider esthetic treatment and physical exercises. |
| Neuropsychiatric changes (sleep disturbances, depression, behavioral, concentration and personality changes) | EFV | Usually self-limited, without need to discontinue ART. Symptomatic treatment, if required.<br>If previous psychiatric disturbance, there is a higher risk of more severe reaction. Effects may be enhanced by alcohol and other psychoactive drugs. |
| Renal Toxicity (nephrolitiasis) | IDV | If using IDV, interrupt IDV and offer hydration, laboratory monitoring and symptomatic treatment (50% recurrence rate). Consider replacing IDV for another PI. |
| Renal Toxicity ( renal tubular dysfunction) | TDF | Discontinue TDF and give supportive treatment. After clinical resolution, resume ART, replacing the offending drug. |
| Peripheral neuropathy | d4T and ddI | Consider replacement by an NRTI with minimal or no neurotoxicity (AZT, TDF or ABC). Symptomatic treatment should be considered. |

# APPENDIX N: CASE REPORT FORMS

List of CRFs

1. Contact Details Form
2. Screening Consent Form
3. Screening Form
4. Enrolment Consent Form
5. Consent for Sample Storage
6. Enrolment Form
7. Randomization Form
8. Nurse Follow up Form
9. Doctor Follow up Form
10. Adherence Form
11. Hematological Toxicity Form
12. Adverse Event Form
13. Death Form
14. Prescription Form
15. Tracing Form
16. Drug log/Register
17. Laboratory Requests Form
18. Consent Withdrawal Form
19. Endpoints Form
20. Hypersensitivity Form
